# Engineering T cells with a membrane-tethered version of SLP-76 overcomes antigen-low resistance to CAR T cell therapy

**DOI:** 10.1038/s43018-025-01056-4

**Published:** 2025-10-23

**Authors:** Maria Caterina Rotiroti, Aidan M. Tousley, Hoyin Chu, Marco Herrera-Barrera, Antigoni Manousopoulou, Won-Ju Kim, Yajie Yin, Thomas Spencer Parish, Aniela Mitchell, Malcolm Holterhus, Lea Wenting Rysavy, Guillermo Nicolas Dalton, Katherine Ann Freitas, Gernot Kaber, Korbinian N. Kropp, Christopher A. Klebanoff, Ansuman T. Satpathy, Leo D. Wang, Caleb A. Lareau, Robbie G. Majzner

**Affiliations:** 1 Department of Pediatric Oncology, Dana-Farber Cancer Institute, Boston, MA USA; 2 Department of Genetics, Stanford University, Stanford, CA USA; 3 Department of Medicine, Stanford University School of Medicine, Stanford, CA USA; 4 Computational and Systems Biology Program, Memorial Sloan Kettering Cancer Center, New York, NY USA; 5 Proteas Health, Torrance, CA USA; 6 Department of Pathology, Stanford University, Stanford, CA USA; 7 Department of Radiation Oncology and Stanford Cancer Institute, Stanford University School of Medicine, Stanford, CA USA; 8 Immunology Graduate Program, Stanford University School of Medicine, Stanford, CA USA; 9 Center for Cancer Cell Therapy, Stanford Cancer Institute, Stanford University School of Medicine, Stanford, CA USA; 10 Immuno-Oncology Program, Memorial Sloan Kettering Cancer Center, New York, NY USA; 11 Parker Institute for Cancer Immunotherapy, San Francisco, CA USA; 12 Department of Pediatrics, City of Hope National Medical Center, Duarte, CA USA; 13 Department of Immuno-oncology, City of Hope National Medical Center, Duarte, CA USA; 14 Division of Hematology/Oncology, Boston Children’s Hospital, Boston, MA USA; 15 Department of Medical Oncology, Dana-Farber Cancer Institute, Boston, MA USA

**Keywords:** Cancer immunotherapy, Immunotherapy, Cancer

## Abstract

Chimeric antigen receptor (CAR) T cells can mediate durable complete responses in individuals with certain hematologic malignancies, but antigen downregulation is a common mechanism of resistance. Although the native T cell receptor can respond to very low levels of antigen, engineered CARs cannot, likely due to inefficient recruitment of downstream proximal signaling molecules. We developed a platform that endows CAR T cells with the ability to kill antigen-low cancer cells consisting of a membrane-tethered version of the cytosolic signaling adaptor molecule SLP-76 (MT-SLP-76). MT-SLP-76 can be expressed alongside any CAR to lower its activation threshold, overcoming antigen-low escape in multiple xenograft models. Mechanistically, MT-SLP-76 amplifies CAR signaling through recruitment of ITK and PLCγ1. MT-SLP-76 was designed based on biologic principles to render CAR T cell therapies less susceptible to antigen downregulation and is poised for clinical development to overcome this common mechanism of resistance.

## Abstract (web summary)

Majzner and colleagues show that engineering T cells with a membrane-tethered version of the signaling adaptor molecule SLP-76 alongside a chimeric antigen receptor (CAR) enhances CAR T cell activity against low-antigen-density tumors.

## Main

Chimeric antigen receptor (CAR) T cells have altered the treatment paradigm for individuals with relapsed and refractory B cell malignancies^1^. Despite high response rates in B cell acute lymphoblastic leukemia (B-ALL) and large B cell lymphoma, only 30–40% of individuals receiving CAR T cells will ultimately be cured of their disease^2–4^. Cure rates for myeloma are even lower^5^. Therapeutic resistance is common, with antigen remodeling being the most frequently observed mechanism for tumor escape. In some cases, complete loss of the target antigen occurs, whereas in others the antigen is simply downregulated below a threshold required for T cell activation^6–12^. Engineering CAR T cells with the ability to respond to antigen-low tumor cells is an area of active investigation^13–17^.

The T cell antigen receptor (TCR) and associated proximal signaling molecules have evolved to carefully calibrate T cell signaling, allowing for both accurate discrimination between foreign and self-proteins and maximal diversity^18^. CAR T cells were empirically designed to mimic the functional output of TCRs by fusing an antigen recognition domain to CD3ζ, the master switch for T cell signaling^19,20^. However, by omitting much of the signaling chassis, CARs have been rendered unable to effectively respond to antigen-low targets^13,14,21–23^. Although minor architectural changes can tune the sensitivity of a CAR, standard CAR designs result in a threshold for activation that is orders of magnitude higher than the native TCR^13,21–25^.

To leverage the superior sensitivity of TCRs, several groups have developed chimeric receptors that fuse antibody-derived binding domains to components of the TCR complex, thereby enabling HLA-independent target recognition^16,26–28^. Although these receptors display enhanced sensitivity, they often require gene editing to eliminate endogenous TCR chains, which complicates the manufacturing process. Furthermore, in some cases, single-chain variable fragments (scFvs) that are functional in CAR designs fail to trigger T cell activation when integrated into these TCR-based systems^28^. Therefore, there is a need for a platform that can enhance signaling from already engineered and clinically validated CAR constructs.

Phosphoproteomic analyses have identified proximal signaling deficits in CARs compared to TCRs, which may explain their reduced antigen sensitivity^14,22^. We found that CAR architectures with higher activation thresholds display reduced phosphorylation of the downstream proximal signaling network. To circumvent this obstacle, we overexpressed proximal TCR signaling molecules in CAR T cells. Although SLP-76 overexpression resulted in enhanced potency, this boost was insufficient to enable recognition of antigen-low targets. SLP-76 is natively located in the cytosol but, during T cell activation, is recruited to the membrane as part of the LAT signalosome. By engineering a membrane-tethered version of SLP-76 (MT-SLP-76), we improved CAR T cell reactivity to antigen-low tumor cells. In mouse models, MT-SLP-76 rescues the activity of CD22-, CD19- and B cell maturation antigen (BCMA)-targeting CARs against antigen-low tumor cells. This work identifies a critical bottleneck for CAR T cell signaling and delivers a readily translatable approach that can be paired with pre-existing CAR designs to improve clinical outcomes.

## Results

### CAR T cell design alters the signaling response

**Fig. 1:**
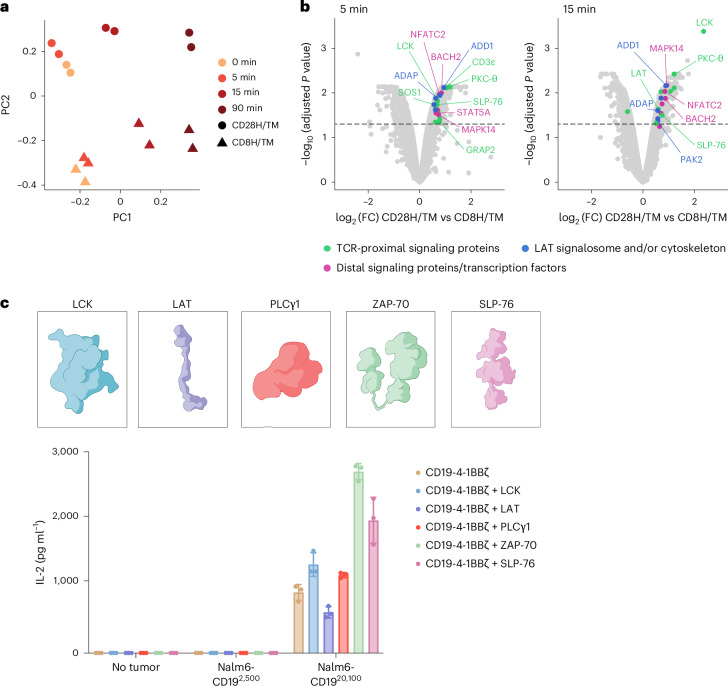
CAR design influences proximal signaling molecule phosphorylation. **a**, CD19-CD8H/TM-4-1BBζ and CD19-CD28H/TM-4-1BBζ CAR T cells from two unique donors were stimulated with 5 μg ml^−1^ anti-CD19 CAR idiotype and goat anti-mouse cross-linking antibodies and incubated at 37 °C for 5, 15 or 90 min. Samples were then processed for proteomic analysis with liquid chromatography–mass spectrometry (LC–MS). PC analysis of quantified phosphopeptides for CD19-CD8H/TM-4-1BBζ and CD19-CD28H/TM-4-1BBζ CAR T cells for the two donors is shown. **b**, Volcano plots depicting FC (log_2_ (FC)) and *P* value (–log_10_(adjusted *P* value)) for differentially phosphorylated peptides identified at 5 and 15 min after stimulation. Differential peptide analyses were conducted using an empirical Bayes moderated *t-*test, and selected proteins with a log_2_ (FC) of ≥0.5 and Benjamini–Hochberg-adjusted *P* value of ≤0.05 are labeled. Green indicates TCR-proximal signaling proteins, blue indicates proteins interacting with the LAT signalosome and/or cytoskeleton, and magenta indicates distal signaling proteins and transcription factors. **c**, Top, schematic of proximal signaling molecules tested for CAR T cell enhancement. Bottom, IL-2 produced by CD19-4-1BBζ CAR T cells with or without overexpression of the indicated proximal signaling molecules after coculture with Nalm6 tumor cells expressing the indicated densities of CD19. Data are shown as mean ± s.d. of three technical replicates and are representative of two independent experiments with different T cell donors. Source data

**Extended Data Fig. 1:**
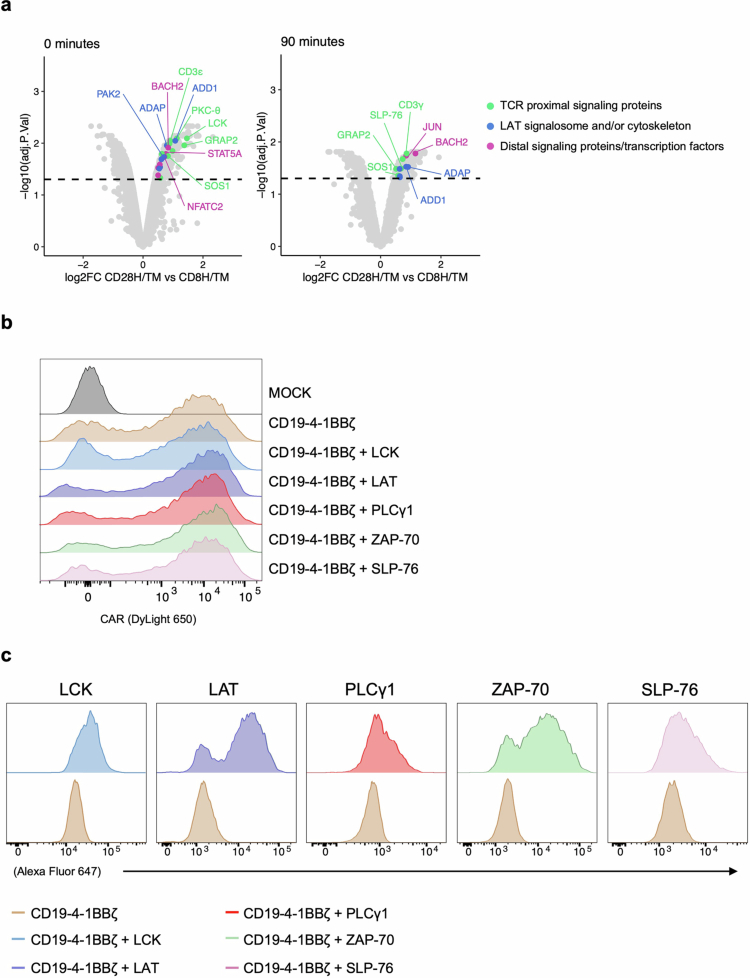
Proteomic and flow cytometric analysis of CARs. **a**, Volcano plots depicting fold change (log_2_FC) and *P* value (-log_10_ adjusted *P* value) for differentially phosphorylated peptides identified in CD19-CD28H/TM-4-1BBζ versus CD19-CD8H/TM-4-1BBζ at 0- and 90-minutes post-stimulation from two unique donors, as described in Fig. 1a. Differential peptide analyses were conducted using an empirical Bayes moderated *T* test, selected proteins with log_2_FC ≥ 0.5, Benjamini-Hochberg adjusted *P* value ≤ 0.05 are labeled. Green indicates TCR proximal signaling proteins, blue indicates proteins interacting with the LAT signalosome and/or cytoskeleton, and magenta indicates distal signaling proteins and transcription factors. **b**, Flow cytometry data showing surface CD19-4-1BBζ CAR expression, and **c**, intracellular staining of proximal signaling molecules in T cells transduced as shown in Fig. 1c, representative of two independent experiments with different T cell donors.

CAR T cells are deficient in recognizing tumor cells expressing low levels of target antigen, providing an opportunity for immune escape through antigen downregulation, as has been observed in clinical trials of CD22 and BCMA CARs^10–12^. The inability of CARs to recognize antigen-low targets has been attributed to impaired recruitment of downstream kinases, such as ZAP-70, and the formation of a poorly organized immune synapse^13,14,22,23,29^. We previously found that CD19 CARs with CD28 rather than CD8 hinge–transmembrane (H/TM) domains display enhanced recognition of antigen-low targets, a finding attributable to improved ZAP-70 recruitment to the immune synapse^13^. We performed quantitative phosphoproteomic analyses comparing CD19 CARs with CD28 versus CD8 H/TM domains after stimulation. Principal component (PC) analysis of the phosphoproteome demonstrated separation of the samples based on duration of stimulation (PC1, 39.9%) and CAR architecture (PC2, 20.1%; Fig. 1a). Comparison of differentially expressed phosphorylated proteins demonstrated enrichment of proximal signaling molecules (LCK, SLP-76, LAT, PKC-θ and CD3 subunits), distal signaling molecules (MAPK) and transcription factors (NFAT, Jun and Bach2) in CD28 H/TM versus CD8 H/TM CAR T cells (Fig. 1b and Extended Data Fig. 1a). Of note, multiple members of the LAT/SLP-76 signalosome^30^, including LAT, SLP-76, GRAP2 and SOS1, were significantly enriched in CD28 H/TM CAR T cells, as were additional proteins linking proximal signaling to cytoskeletal rearrangement (for example, adhesion and degranulation-promoting adaptor protein (ADAP), ADD1 and PAK2). Although many of these phosphoproteins were increased only after antigen encounter, some were phosphorylated before activation, indicating that CAR structure may also prime cells for more efficient antigen recognition. Overall, these findings align with published data demonstrating that superior antigen-low recognition by TCRs versus CARs is linked to enhanced phosphorylation of proximal signaling molecules^14,17,22^.

We next asked whether overexpression of key proximal signaling molecules in CAR T cells could enable response to antigen-low targets. We overexpressed five proximal signaling molecules (LCK, ZAP-70, LAT, SLP-76 and PLCγ1) in CD19 CAR T cells before exposure to antigen-low and antigen-high target cells. Although ZAP-70 and SLP-76 overexpression resulted in enhanced interleukin-2 (IL-2) production in response to antigen-high targets, it did not alter recognition of antigen-low target cells (Fig. 1c and Extended Data Fig. 1b,c).

### Tethering SLP-76 to the membrane restores sensitivity to antigen-low target cells

**Fig. 2:**
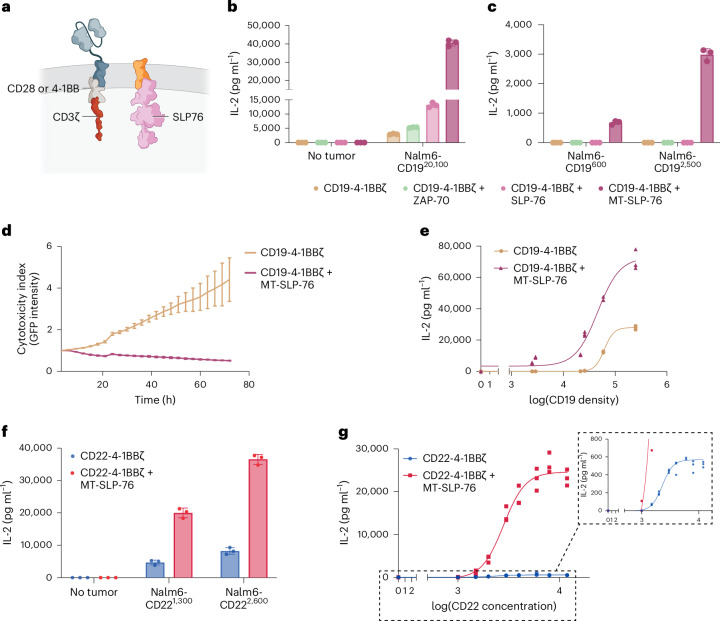
Tethering SLP-76 to the membrane lowers CAR T cell activation threshold. **a**, Schematic of membrane-tethered SLP-76 (MT-SLP-76). **b**,**c**, IL-2 produced by CD19-4-1BBζ CAR T cells with or without overexpression of the indicated proximal signaling molecules after coculture with Nalm6 tumor cells expressing various densities of CD19. Data are shown as mean ± s.d. of three technical replicates and are representative of four independent experiments with different T cell donors. **d**, Killing of Nalm6-CD19^600^ tumor cells by CD19-4-1BBζ CAR T cells ± MT-SLP-76, measured as relative intensity of green fluorescence over 72 h with the Incucyte live-cell analysis system and normalized to *t* = 0. Data are shown as mean ± s.d. of three technical replicates and are representative of eight independent experiments with four different T cell donors. **e**, IL-2 production by CD19-4-1BBζ CAR T cells ± MT-SLP-76 cocultured with a library of Nalm6 clones expressing different densities of CD19. Shown is the concentration of IL-2 measured as a function of log (CD19 molecule number) for that specific clone, with three technical replicates included. Curve fitting was performed using a four-parameter variable-slope dose–response curve. Data are representative of two independent experiments with different T cell donors. **f**, IL-2 produced by CD22-4-1BBζ CAR T cells ± MT-SLP-76 cocultured with Nalm6 cells expressing endogenous or low levels of CD22. Data are shown as mean ± s.d. of three technical replicates and are representative of six independent experiments with three different T cell donors. **g**, IL-2 produced by CD22-4-1BBζ CAR T cells ± MT-SLP-76 after stimulation with plate-bound recombinant CD22 protein. Shown is the concentration of IL-2 measured as a function of log (recombinant CD22 concentration), with three technical replicates included. Curve fitting was performed using a four-parameter variable-slope dose–response curve. Data are representative of two independent experiments with different T cell donors. Source data

**Extended Data Fig. 2:**
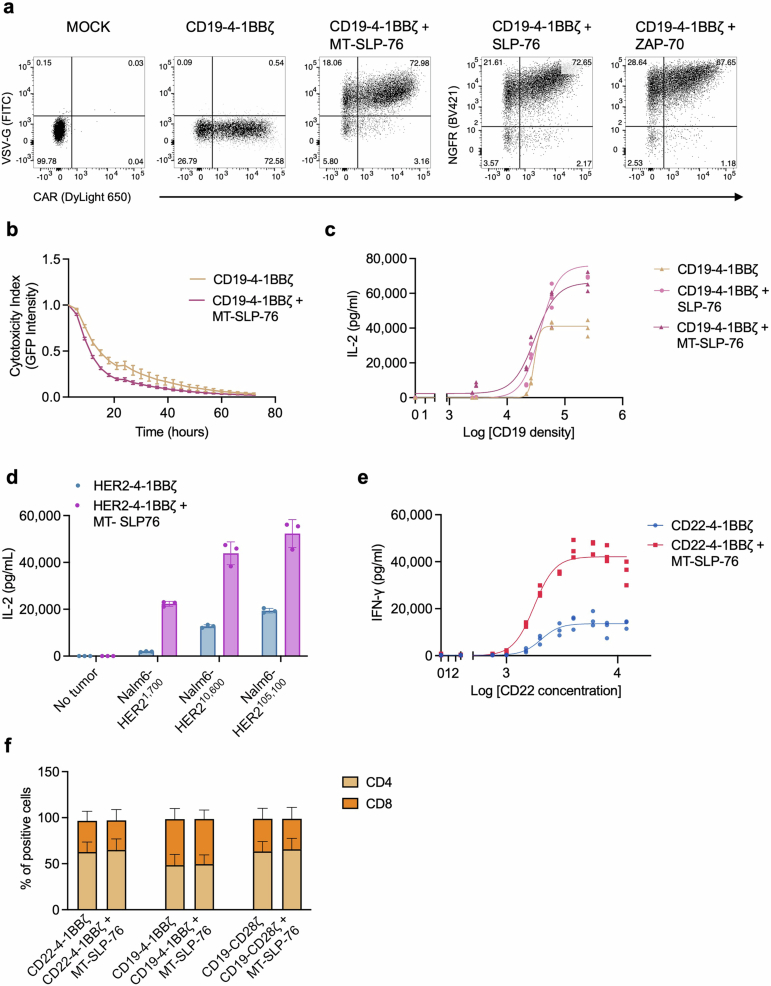
MT-SLP-76 enhances CAR T cell activity *in vitro.* **a**, Flow cytometric data showing co-expression of CD19 CAR and the indicated proximal signaling molecules detected via surface marker staining (VSV-G tag for MT-SLP-76; truncated NGFR for cytosolic molecules), representative of four independent experiments with different T cell donors. **b**, Killing of Nalm6-CD19^20,100^ by CD19-4-1BBζ CAR T cells ± MT-SLP-76, measured as relative intensity of green fluorescence over 72 hours with the Incucyte live-cell analysis system and normalized to t = 0. Data are mean ± S.D. of three technical replicates, representative of eight independent experiments with four different T cell donors. **c**, IL-2 production by CD19-4-1BBζ CAR T cells ± SLP-76 or MT-SLP-76 co-cultured with a library of Nalm6 clones expressing different densities of CD19. Shown is the concentration of IL-2 measured as a function of log of the CD19 molecule number for that specific clone, with three technical replicates included. Curve fitting was performed using a four-parameter variable slope dose–response curve. Data are representative of two independent experiments with different T cell donors. **d**, IL-2 produced by HER2-4-1BBζ CAR T cells ± MT-SLP-76 after co-culture with Nalm6 clones expressing various levels of HER2. Data are mean ± S.D. of three technical replicates, representative of two independent experiments with different T cell donors. **e**, IFN-γ produced by CD22-4-1BBζ CAR T cells ± MT-SLP-76 after stimulation with plate-bound recombinant CD22 protein. Shown is the concentration of IFN-γ measured as a function of log of the recombinant CD22 concentration, with three technical replicates included. Curve fitting was performed using a four-parameter variable slope dose-response curve. Representative of two experiments with different T cell donors. **f**, Analysis of CD4 and CD8 percentage in T cells expressing the specified CARs ± MT-SLP-76. Data are mean ± S.D. of n = 6 for CD22-4-1BBζ, n = 4 for CD19-4-1BBζ and n = 2 for CD19-CD28ζ. Source data

As CARs are deficient in recruiting proximal signaling molecules to the immune synapse, we hypothesized that tethering a signaling molecule to the cell membrane could facilitate its engagement and result in enhanced activity. Although we were unable to express a full-length membrane-tethered ZAP-70 in T cells^31^, we successfully engineered a membrane-tethered version of SLP-76 (MT-SLP-76; Fig. 2a) that is efficiently expressed by T cells (Extended Data Fig. 2a). Coexpression of MT-SLP-76 alongside a CD19-4-1BBζ CAR substantially enhanced IL-2 production in response to both CD19-high and CD19-low target cells compared to overexpression of either native SLP-76 or ZAP-70 (Fig. 2b,c and Extended Data Fig. 2a). MT-SLP-76 considerably improved killing of CD19-low cells, while maintaining similar efficacy against antigen-high targets (Fig. 2d and Extended Data Fig. 2b). We evaluated CD19 CAR activity with or without MT-SLP-76 against cell lines engineered to express a range of CD19 antigen densities (600 to 249,700 molecules per cell; Supplementary Table 1)^13^. MT-SLP-76 shifted the antigen density response curve, lowering the threshold for cytokine secretion compared to the CD19-4-1BBζ CAR (Fig. 2e). We also observed a lower threshold for antigen recognition when comparing overexpression of MT-SLP-76 to native, cytosolic SLP-76 (Extended Data Fig. 2c). Overexpression of MT-SLP-76 also conferred CD22- and HER2-targeting CAR T cells with enhanced sensitivity against antigen-low and antigen-high target cells (Fig. 2f and Extended Data Fig. 2d), similarly shifting the cytokine response curve (Fig. 2g and Extended Data Fig. 2e), establishing the generalizability of this approach. MT-SLP-76 overexpression altered the cytokine response of CAR T cells without impacting their CD4:CD8 ratio (Extended Data Fig. 2f).

### MT-SLP-76 enhances CAR T cell activity in vivo

**Fig. 3:**
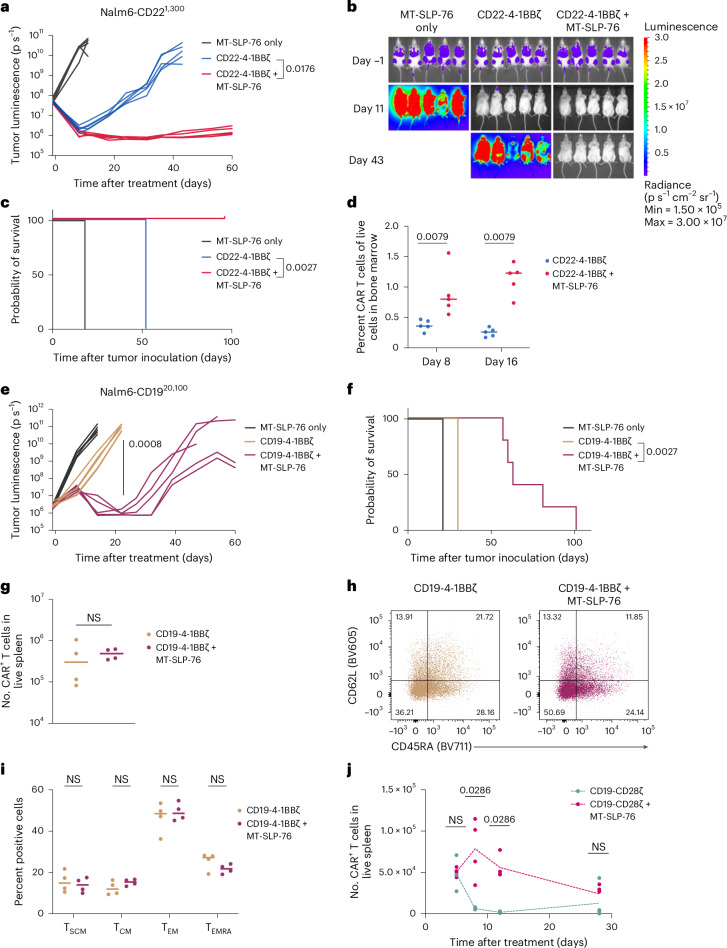
MT-SLP-76 overcomes antigen-low escape in vivo*.* **a**, Mice were treated with 6 × 10^6^ CD22-4-1BBζ CAR T cells ± MT-SLP-76 or control cells (expressing MT-SLP-76 only) 3 days after inoculation with 1 × 10^6^ Nalm6-CD22^1,300^ cells. Shown is the quantification of tumor progression for each individual mouse as measured by photon flux values determined by bioluminescence imaging (BLI); *n* = 5 mice per group. **b**,**c**, Representative bioluminescence images (**b**) and survival curves (**c**) of mice treated as in **a**. Data in **a**–**c** are representative of four independent experiments with different T cell donors. **d**, Percentage of CD22 CAR T cells detected by flow cytometry in the bone marrow of mice treated as in **a** on days 8 and 16 after treatment; *n* = 5 mice per group. **e**, Mice were treated with 1 × 10^6^ CD19-4-1BBζ CAR T cells ± MT-SLP-76 or control cells 3 days after inoculation with 1 × 10^6^ million Nalm6-CD19^20,100^ (wild-type Nalm6) cells. Shown is the quantification of tumor progression for each individual mouse as measured by photon flux values determined by BLI; *n* = 5 mice per group. **f**, Survival curves of mice treated as in **e**. **g**, Mice were treated with 7 × 10^6^ CD19-4-1BBζ CAR T cells ± MT-SLP-76 cells 3 days after inoculation with 1 × 10^6^ Nalm6-CD19^20,100^ cells. Twenty-one days after treatment, spleens were collected, and CAR T cell persistence was assessed by flow cytometry. Data are shown as mean ± s.e.m. of *n* = 4 mice. **h**,**i**, Representative flow cytometric plots (**h**) and quantification (**i**) of CAR T stem cell memory T (T_SCM_) cells (CD62L^+^CD45RA^+^), central memory T (T_CM_) cells (CD62L^+^CD45RA^−^), effector memory T (T_EM_) cells (CD62L^−^CD45RA^−^) and terminal effector memory T (T_EMRA_) cells (CD62L^−^CD45RA^+^); *n* = 4 mice per group. **j**, Mice were treated with 5 × 10^6^ CD19-CD28ζ CAR T cells ± MT-SLP-76 3 days after inoculation with 1 × 10^6^ Nalm6-CD19^20,100^ cells. Absolute CAR T cell numbers in the spleen were measured by flow cytometry on days 5, 8, 12 and 28 after treatment (*n* = 4 mice per time point). Statistical comparisons were performed using repeated-measures two-way analysis of variance (ANOVA) with correction for multiple comparisons (**a** and **e**), pairwise log-rank tests without correction for multiple comparisons (**c** and **f**), two-way ANOVA (**i**) or Mann–Whitney test (two-tailed; **d**, **g** and **j**); NS, not significant. Source data

**Extended Data Fig. 3:**
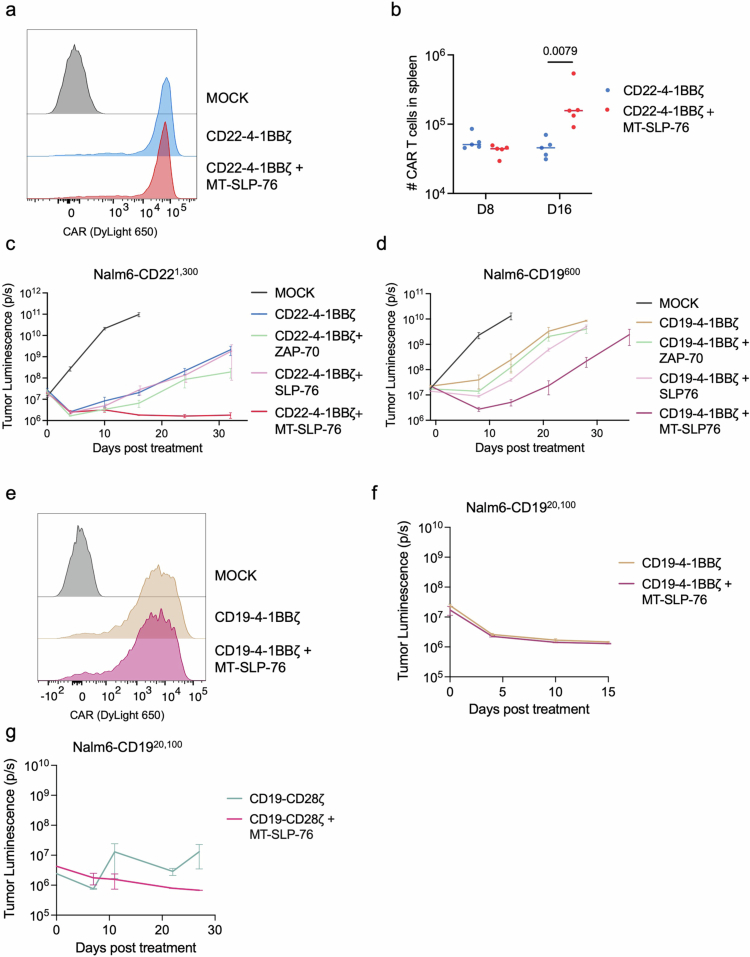
MT-SLP-76 enhances CAR T cell activity *in vivo.* **a**, Flow cytometric data showing CD22-4-1BBζ expression on T cells from experiments described in Fig. 3a, representative of four independent experiments. **b**, Absolute CD22 CAR T cell numbers detected in the spleen of mice from experiment described in Fig. 3a. Statistical analysis was performed with the Mann–Whitney test, n = 5 mice per group. **c**, Mice were treated with 7×10^6^ CD22-4-1BBζ CAR T cells ± the indicated proximal signaling molecules or control cells (MOCK) three days after inoculation with 1×10^6^ Nalm6-CD22^1,300^. Shown is the quantification of tumor progression determined by bioluminescence imaging (BLI). Data are mean ± S.E.M. of n = 6 mice for CD22-4-1BBζ + MT-SLP-76 and n = 5 mice for all other groups. Representative of two independent experiments with different T cell donors. **d**, Mice were treated with 3×10^6^ CD19-4-1BBζ CAR T cells ± the indicated proximal signaling molecules or MOCK four days after inoculation with 1×10^6^ Nalm6-CD19^600^ cells. Shown is the quantification of tumor progression determined by BLI. Data are mean ± S.E.M. of n = 6 mice for CD19-4-1BBζ + ZAP-70 and n = 5 mice for all other groups. **e**, Flow cytometric data showing CD19-4-1BBζ CAR expression on T cells from one experiment described in Fig. 3e-f. **f**, Tumor quantification by BLI of mice treated in experiment described in Fig. 3g. Data are mean ± S.E.M. of n = 4 mice. **g**, Tumor quantification by BLI of mice treated in experiment described in Fig. 3j. Data are mean ± S.E.M. of n = 4 mice. Source data

CD22 downregulation is a primary cause of CD22 CAR resistance in children with B-ALL. We therefore next tested MT-SLP-76 in a previously described model of CD22-low leukemia (1,300 molecules per cell) in which CD22 CAR T cells fail to control antigen-low disease^10^. MT-SLP-76-overexpressing CD22 CAR T cells mediated sustained tumor eradication, whereas CD22 CAR T cells alone induced only modest tumor control (Fig. 3a–c and Extended Data Fig. 3a). It was previously shown that CD22 CAR T cells expand poorly in response to the CD22-low tumor cells in this model^32^. MT-SLP-76 rescued CAR T cell expansion in vivo, resulting in significantly increased CAR T cell numbers in the bone marrow and spleens of treated mice (Fig. 3d and Extended Data Fig. 3b). These data demonstrate that MT-SLP-76 can restore CD22 CAR activity in a clinically relevant model of antigen-low B-ALL. To confirm the necessity of tethering SLP-76 to the membrane versus overexpression of cytosolic proximal signaling molecules in vivo, we compared MT-SLP-76 to overexpression of cytosolic SLP-76 or ZAP-70 in this model and found that only MT-SLP-76 restored functionality against CD22-low leukemia (Extended Data Fig. 3c). Similarly, MT-SLP-76 enhanced in vivo activity of CD19-4-1BBζ CAR in a model of CD19 ultra-low leukemia (600 molecules cell^−^^1^), whereas overexpression of either cytosolic SLP-76 or ZAP-70 had minimal effect (Extended Data Fig. 3d).

Boosting signal strength could potentially lead to overactivation against antigen-high targets, which could theoretically result in reduced T cell persistence and/or diminished tumor control in vivo. Therefore, we assessed MT-SLP-76 activity in models of wild-type CD19-high Nalm6 (Nalm6-CD19^20,100^) cells. First, in a stress test model, we observed significantly enhanced antitumor activity and survival from a CD19-4-1BBζ CAR when coexpressed with MT-SLP-76 (Fig. 3e,f and Extended Data Fig. 3e). To assess whether MT-SLP-76 could negatively affect CAR T cell persistence, we treated mice bearing Nalm6 wild-type xenografts with curative doses of CD19-4-1BBζ CAR T cells (Extended Data Fig. 3f). After 21 days, mice treated with CD19-4-1BBζ + MT-SLP-76 CAR T cells displayed equivalent persistence and a similar memory phenotype as those treated with CD19-4-1BBζ CAR alone (Fig. 3g–i). To confirm that MT-SLP-76 expression does not result in unchecked proliferation, we tracked CD19-CD28ζ CAR T cells with or without MT-SLP-76 in an additional curative model (Extended Data Fig. 3g) at multiple time points. Despite reaching higher peak CAR T cell numbers, the CD19-CD28ζ + MT-SLP-76 CAR T cells contracted to similar numbers as in the CAR-alone condition (Fig. 3j). These data indicate that CAR T cells bearing MT-SLP-76 demonstrate enhanced antitumor activity against a broad range of antigen densities without compromising the capacity for CAR T cell persistence.

### Antigen selection for MT-SLP-76

**Fig. 4:**
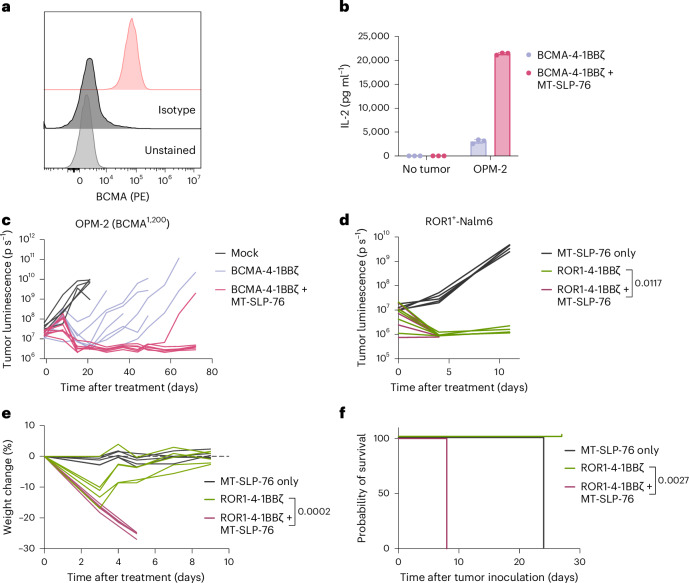
MT-SLP-76 lowers the CAR T cell activation threshold, altering the therapeutic window for certain antigen targets. **a**, Flow cytometric analysis showing expression of BCMA on the OPM-2 multiple myeloma cell line; data are representative of two independent experiments. **b**, IL-2 production by BCMA-4-1BBζ CAR T cells ± MT-SLP-76 after coculture with OPM-2 cells. Data are shown as mean ± s.d. of three technical replicates; data are representative of four independent experiments with different T cell donors. **c**, Mice were treated with 0.4 × 10^6^ BCMA-4-1BBζ CAR T cells ± MT-SLP-76 or control mock cells 3 weeks after inoculation with 1 × 10^6^ OPM-2 cells. Shown is the quantification of tumor progression for each individual mouse determined by BLI; *n* = 5 mice for mock, *n* = 7 for BCMA CAR and *n* = 8 for BCMA CAR + MT-SLP-76. **d**, Mice were treated with 5 × 10^6^ ROR1-4-1BBζ CAR T cells ± MT-SLP-76 or MT-SLP-76-only control cells 3 days after inoculation with 1 × 10^6^ ROR1^+^-Nalm6 tumor cells. Shown is the quantification of tumor progression for each individual mouse determined by BLI. **e**, Weight of individual mice treated as in **d** shown as the percent change from initial weight. **f**, Survival curves of mice treated as in **d**; *n* = 5 mice per group. Statistical comparisons were performed using a repeated-measures two-way ANOVA with correction for multiple comparisons (**d** and **e**) or pairwise log-rank tests without correction for multiple comparisons (**f**). Source data

**Extended Data Fig. 4:**
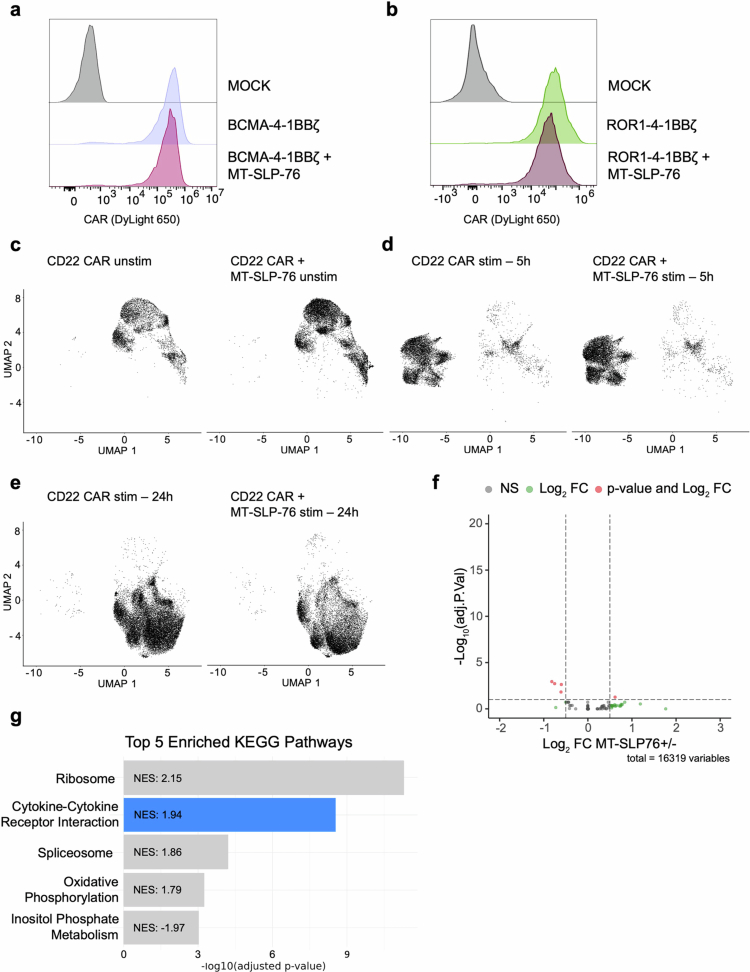
CAR expression and transcriptomic profiling. **a**, Flow cytometric data showing BCMA-4-1BBζ CAR expression on the T cells from one experiment described in Fig. 4c. **b**, Flow cytometric data showing ROR1-4-1BBζ CAR expression on the T cells from one experiment described in Fig. 4d-f. **c-e**, UMAP visualizations from single-cell RNA sequencing experiment described in Fig. 5a–e showing CD22-4-1BBζ CAR T cells ± MT-SLP-76 in separate panels at baseline (**c**), after 5 hours (**d**), and after 24 hours (**e**) of stimulation, from two donors. **f**, Volcano plots of log_2_FC and log_10_ Benjamini-Hochberg adjusted *P* value for the differentially expressed genes in CD22-4-1BBζ CAR T cells ± MT-SLP-76 without stimulation as determined by the Wald test using DESeq2. **g**, Bar chart showing the top five Kyoto Encyclopedia of Genes and Genomes (KEGG) pathways in CD22-4-1BBζ CAR T cells with MT-SLP-76 versus CD22-4-1BBζ CAR T cells after 5 h of stimulation. Pathway enrichment analysis was performed using gene set enrichment analysis (GSEA) as implemented by fast GSEA. Pathways are ranked by –log₁₀ Benjamini–Hochberg adjusted *P* value, and the normalized enrichment score (NES) is shown for each term.

Given that BCMA downregulation has also been reported as a mechanism of resistance in clinical trials^11,12^, we aimed to validate MT-SLP-76 in a model of multiple myeloma with low BCMA expression (OPM-2 cells, 1,200 molecules per cell; Fig. 4a). MT-SLP-76 resulted in higher IL-2 production in response to antigen encounter (Fig. 4b), which translated to substantially improved antitumor activity in vivo (Fig. 4c and Extended Data Fig. 4a), demonstrating that this approach has applicability across disease types.

Although these data could serve as a rationale for clinically testing BCMA CAR + MT-SLP-76, it is important to note that BCMA may also be expressed at lower levels on the basal ganglia, which may be the cause of a Parkinson’s-like movement disorder observed in selected individuals treated with BCMA CAR T cells^33,34^. Although enhanced sensitivity may be desirable for targeting antigens with limited normal tissue liabilities, such as CD19 and CD22, this could also narrow the therapeutic window for antigens with low-level expression on normal, vital tissues. To explore this concept, we used a previously published model in which a cross-reactive ROR1 CAR causes on-target, off-tumor toxicity (OTOTT) due to the recognition of ROR1 on normal tissues^35^. In the published model, this CAR mediated lethal OTOTT only after preconditioning (radiation or chemotherapy), whereas mice treated without preconditioning recovered from transient toxicity^35^. We treated mice bearing ROR1^+^-Nalm6 xenografts with T cells expressing this ROR1 CAR ± MT-SLP-76 without any preconditioning. ROR1 CAR T cells with or without MT-SLP-76 eradicated all tumor cells (Fig. 4d and Extended Data Fig. 4b). Although mice that received ROR1 CAR T cells experienced transient weight loss from which they recovered, those treated with ROR1 CAR + MT-SLP-76 T cells continued to lose weight due to OTOTT, necessitating euthanasia within 1 week (Fig. 4e,f). Therefore, although MT-SLP-76 overexpression is a promising strategy to target tumor cells with low antigen expression, caution is warranted with antigens that may be expressed at low levels on normal tissue, which may require Boolean logic gating for safe and effective targeting^31,36–39^.

### MT-SLP-76 specifically drives cytokine and chemokine signatures

**Fig. 5:**
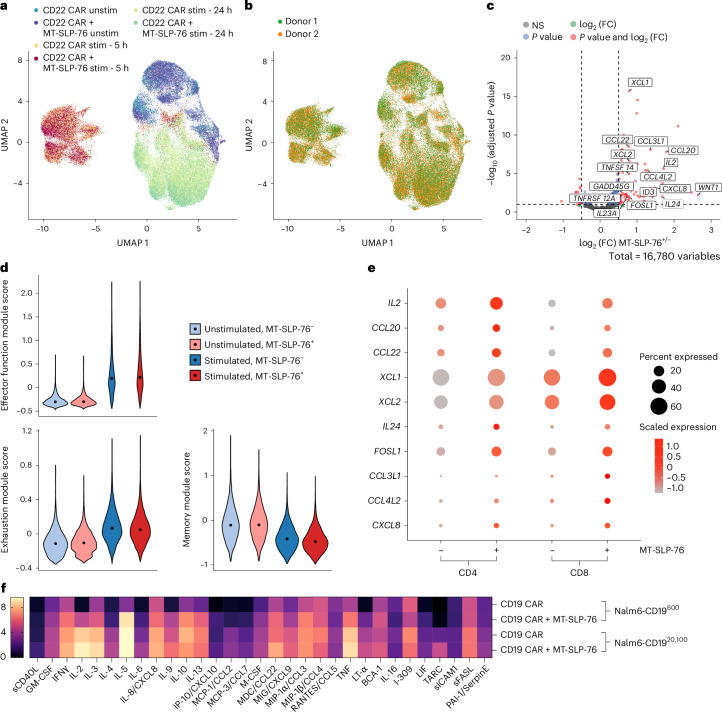
MT-SLP-76 enhances cytokine/chemokine responses. **a**, CD22-4-1BBζ CAR T cells ± MT-SLP-76 were stimulated with Nalm6-CD22^1,300^ cells for 5 or 24 h and subjected to single-cell RNA sequencing. Shown is a uniform manifold approximation and projection (UMAP) embedding visualization with overlaid CD22-4-1BBζ ± MT-SLP-76 CAR T cells ± stimulation; *n* = 2 donors; stim, stimulated; unstim, unstimulated. **b**, UMAP embedding visualization showing an overlay annotating two T cell donors. **c**, Volcano plot of log_2_ (FC) and log_10_ (adjusted *P* value) for differentially expressed genes after 5 h of stimulation. Selected genes with a log_2_ (FC) of ≥0.5 and Benjamini–Hochberg adjusted *P* value of ≤0.05, as determined by the Wald test using DESeq2, are labeled. **d**, Violin plots characterizing single-cell gene expression modules for T cell effector function (top left; *n* = 18 genes), T cell exhaustion (bottom left; *n* = 9 genes) and T cell memory (right; *n* = 7 genes) signatures in CD22-4-1BBζ CAR T cells ± MT-SLP-76 CAR T cells ± stimulation. **e**, Dot plot of selected cytokine- and chemokine-related genes in CD4^+^ and CD8^+^ CD22-4-1BBζ CAR T cells ± MT-SLP-76 after stimulation. Color represents normalized expression level, and dot size represents expression percentage. **f**, Heat map generated from Luminex multiplex cytokine analysis of supernatants from CD19-4-1BBζ CAR T cells ± MT-SLP-76 cocultured with Nalm6-CD19^600^ or Nalm6-CD19^20,100^ cells. Average mean fluorescence intensity (MFI) results are represented as log_2_ (FC) from the unstimulated conditions. Shown are the cytokines reaching a log_2_ (FC) of ≥1.5 in at least one condition. Data are from one experiment; sCD40L, soluble CD40L; GM-CSF, granulocyte–macrophage colony-stimulating factor; IFNγ, interferon-γ; IP-10, IFNγ-induced protein 10; MCP, monocyte chemoattractant protein; M-CSF, macrophage colony-stimulating factor; MDC, macrophage-derived chemokine; MIG, monokine induced by IFNγ; MIP, macrophage inflammatory protein; BCA, B cell-attracting chemokine; LIF, leukemia inhibitory factor; TARC, thymus and activation-regulated chemokine; sICAM-1, soluble intercellular adhesion molecule 1; sFASL, soluble Fas ligand; PAI-1, plasminogen activator inhibitor 1. Source data

To delineate the effects of MT-SLP-76 on T cell transcriptional programs, we stimulated CD22 CAR T cells ± MT-SLP-76 for 5 or 24 h with CD22-low Nalm6 cells and subjected them to single-cell RNA sequencing. CD22 CAR T cells clustered largely by antigen exposure and not whether they expressed MT-SLP-76 (Fig. 5a,b and Extended Data Fig. 4c–e). After 5 h of stimulation, we identified only 72 genes that were significantly upregulated (63 genes) or downregulated (9 genes) with a ≥0.5 log_2 _(fold change) (log_2_ (FC)) between the two groups (Fig. 5c and Extended Data Fig. 4f). Overall T cell effector function, exhaustion and memory scores were nearly identical between antigen-challenged CD22 CAR T cells ± MT-SLP-76 (Fig. 5d). By 24 h after stimulation, the transcriptional programs of CD22 CARs with or without MT-SLP-76 more closely resembled those of unstimulated CAR T cells (Extended Data Fig. 4c–e). These modest transcriptional changes contrast with many published mechanisms for enhancing CAR T cell functionality through genetic or transcriptional reprogramming, which frequently result in hundreds or thousands of differentially expressed genes^40–44^. Together, our transcriptome-wide expression analyses suggest that MT-SLP-76 overexpression may enhance CAR T cells in a more targeted capacity than other modifications that result in large-scale CAR T cell state changes.

In evaluating genes that were differentially expressed, we found enrichment for those involved in cytokine and chemokine signaling by gene pathway analysis (Extended Data Fig. 4g), including *IL2*, *CCL3L1*, *IL8* (*CXCL8*), *XCL1/XCL2* and *FOSL1* (Fig. 5e). These data align with findings linking SLP-76 activity to cytokine production (for example, IL-2) in activated T cells^45^. Quantitative measurement by Luminex confirmed enhanced cytokine and chemokine production by CAR T cells with MT-SLP-76, including IL-2, TNF, CXCL8 and CCL22 (Fig. 5f).

### MT-SLP-76 activity depends on ITK and PLCγ1 recruitment

**Fig. 6:**
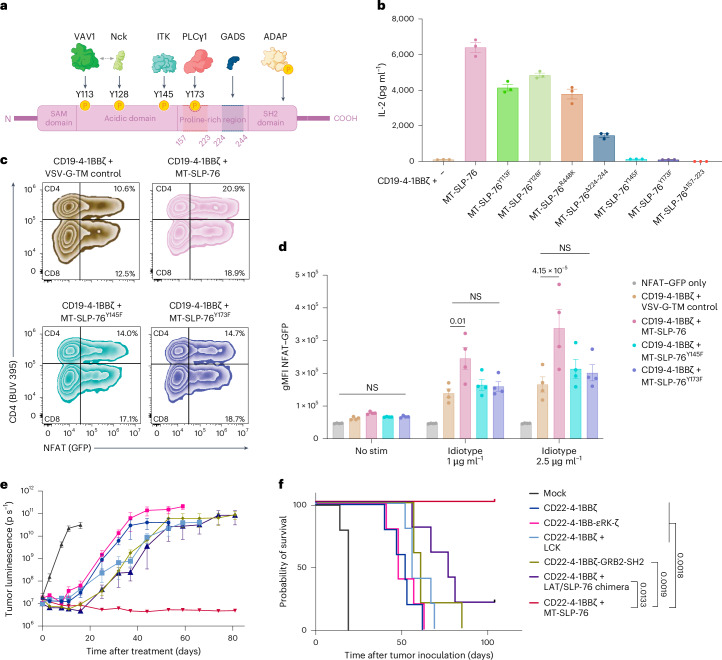
MT-SLP-76 signaling is dependent on ITK and PLCγ1. **a**, Schematic illustrating SLP-76 and binding/interaction sites for associated proteins. Tyrosines 113 and 128, once phosphorylated, become docking sites for the guanine nucleotide exchange factor VAV1 and the adaptor Nck^47^. Phosphorylated tyrosine 145 allows for SLP-76 association with ITK^47,52^. The 157–223 region of SLP-76 is required for PLCγ1 binding, with phosphorylation of tyrosine 173 required for PLCγ1 activation^51^. The 224–244 region encompasses the GADS binding site, involved in the interaction between SLP-76 and LAT^49^. Arginine 448 within the SLP-76 SH2 domain, is critical for the association of SLP-76 with ADAP^48^. **b**, IL-2 production by CD19-4-1BBζ CAR T cells ± MT-SLP-76 bearing the indicated mutations or deletions after coculture with Nalm6-CD19^2,500^ cells. Data are shown as mean ± s.d. of three technical replicates and are representative of three independent experiments with different T cell donors. **c**, CD19-4-1BBζ CAR T cells ± MT-SLP-76 bearing the indicated mutations and transduced with an NFAT–GFP reporter were stimulated for 6 h with varying concentrations of anti-CD19 CAR idiotype and goat anti-mouse cross-linking antibodies. Cells were then analyzed by flow cytometry for CAR, VSV-G tag, CD4, CD8 and GFP expression. Representative plots show GFP expression in CD4⁺ and CD8⁺ CAR T cells (gated on CAR⁺, VSV-G⁺ cells) at the 2.5 μg ml^−^^1^ stimulation condition. **d**, Geometric MFI (gMFI) of NFAT-induced GFP expression was quantified within the GFP⁺ population of responding cells. Data are shown as mean ± s.e.m. from four different T cell donors across two experiments, each tested in three technical replicates. Statistical analysis was performed using two-way ANOVA. **e**, Mice were treated with 5 × 10^6^ of the indicated CD22 CAR constructs or control T cells (mock) 3 days after inoculation with 1 × 10^6^ Nalm6-CD22^1,300^ cells. Shown is the quantification of tumor progression determined by BLI. Data are shown as mean ± s.e.m. of *n* = 5 mice per group. **f**, Survival curves of mice treated as in **e**. Comparisons were performed using pairwise log-rank tests without correction for multiple comparisons. Data in **e** and **f** are representative of two independent experiments with different T cell donors. Source data

**Extended Data Fig. 5:**
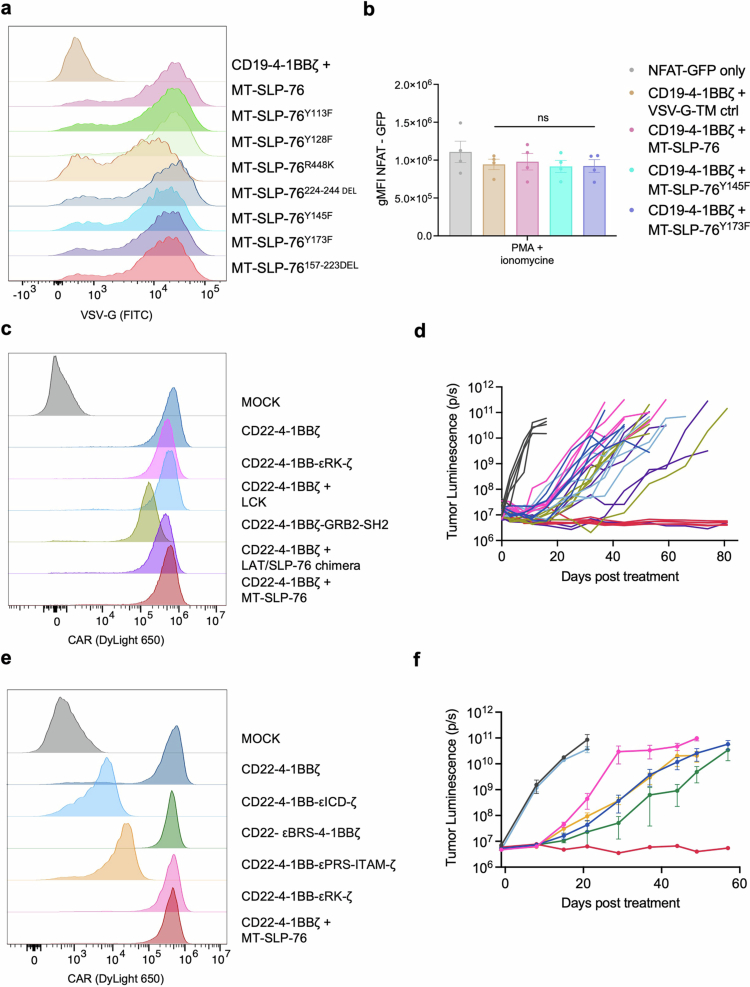
MT-SLP-76 outperforms other methods for utilizing proximal signaling molecules for CAR enhancement. **a**, Flow cytometric analysis of surface tag VSV-G on CAR T cells as a measure of MT-SLP-76 expression from experiments described in Fig. 6a-b, representative of three independent experiments with different T cell donors. **b**, Geometric mean fluorescence intensity (gMFI) of NFAT-induced GFP expression following 6 hour stimulation with PMA and ionomycin was quantified within the GFP⁺ population of responding cells from experiment described in Fig. 6c-d. Data are presented as mean ± S.E.M. from four independent T cell donors across two experiments, each tested in three technical replicates. Statistical analysis was performed using one-way ANOVA. **c**, Flow cytometric analysis of CD22 CAR expression on T cells from experiments described in Fig. 6e-f, representative of two independent experiments with different T cell donors. **d**, Quantification of tumor progression by BLI for each individual mouse for the experiment described in Fig. 6e, n = 5 mice per group. **e**, Flow cytometric analysis of indicated CD22 CAR constructs integrating portions of CD3ε in the endodomains, from one experiment. **f**, Mice were inoculated with 1×10^6^ Nalm6-CD22^1,300^ and then treated with 5×10^6^ of indicated CAR T cells. Shown is the quantification of tumor progression determined by BLI. Data are mean ± S.E.M. of n = 5 mice, n = 4 for MOCK. Source data

We next sought to establish the molecular mechanism by which MT-SLP-76 enhances CAR T cell function. SLP-76 is a multidomain scaffold that links proximal and distal signaling events and is responsible for the recruitment of multiple proteins including VAV1, noncatalytic region of tyrosine kinase (Nck), IL-2-inducible T cell kinase (ITK), ADAP, GADS, LAT and PLCγ1 (ref. ^46^). To identify the key molecules involved in MT-SLP-76-mediated enhancement, we generated versions of MT-SLP-76 bearing mutations or deletions inactivating the function of specific SLP-76 domains (Fig. 6a and Extended Data Fig. 5a)^47–52^. We challenged CD19-4-1BBζ CAR T cells with or without MT-SLP-76 variants with CD19-low Nalm6 cells. Preventing MT-SLP-76 association with the proteins VAV1 (Y113F), Nck (Y128F), ADAP (R448K) or GADS/LAT (deletion of amino acids 224–244) did not abrogate MT-SLP-76 enhancement (Fig. 6b). By contrast, MT-SLP-76 enhancement was eliminated by mutating either the tyrosine required for ITK recruitment and activation (Y145F) or the tyrosine phosphorylated by ITK (Y173F), both of which are required for the ultimate PLCγ1 activation. Similarly, a deletion of the proline-rich region of SLP-76 (157–223) that abolishes its association with PLCγ1 also eliminated MT-SLP-76-based enhancement. These results demonstrate that MT-SLP-76-mediated enhancement relies on PLCγ1 activation through ITK. We evaluated the downstream signaling effects of PLCγ1 activation by measuring NFAT transcriptional activity using an NFAT-inducible green fluorescent protein (GFP) reporter system^48,51^. Following idiotype stimulation, MT-SLP-76-expressing CD19-4-1BBζ CAR T cells elicited significantly stronger NFAT–GFP responses than controls, including MT-SLP-76 with Y145F or Y173F point mutations (Fig. 6c,d and Extended Data Fig. 5b), indicating more robust signal activation.

### MT-SLP-76 outperforms other proximal signaling enhancements

Several groups have used proximal signaling molecules to enhance engineered T cell activity^14,53–55^. Many of these approaches rely on integration of additional signaling molecules into the CAR itself, which can complicate engineering and receptor expression. Only some of these maneuvers have been tested in models of low antigen density. We compared the efficacy of MT-SLP-76 to some of these approaches, including incorporation of CD3ε or GRB2 (an adapter molecule that links LAT and SLP-76)^14^ domains into the CAR and overexpression of LCK^53^ or a LAT/SLP-76 chimera^49^ in the CD22-low Nalm6 cell model. For CD3ε, several groups have identified its importance in TCR versus CAR signaling^14,54,55^, and we tested integration of multiple different CD3ε endodomains into the CAR. Compared to the other CAR structures and overexpression conditions, only MT-SLP-76 eradicated antigen-low tumors, resulting in long-term survival of treated mice (Fig. 6e,f and Extended Data Fig. 5c–f). Thus, MT-SLP-76 may be uniquely suited for clinical deployment to overcome antigen-low escape.

## Discussion

CAR T cells mediate durable remissions in hematologic malignancies, including B cell leukemia, lymphoma and myeloma, but therapeutic resistance has emerged through antigen remodeling^9–12,56^. In some cases, tumor cells exhibit complete antigen loss, whereas in others they downregulate the target antigen below a threshold necessary for CAR T cell activation^6–12,56^. This mechanism of resistance is surprising given that the native TCR can respond to as low as one to ten peptides presented in major histocompatibility complex^57,58^.

TCR signaling is carefully orchestrated to allow specific and potent recognition of foreign or mutated peptides without permitting response to low-affinity self-peptides, all while maintaining a degree of cross-reactivity to increase diversity^59,60^. Although CAR T cells harness the same signaling networks as the TCR^31^, activation occurs in response to ligation of an scFv that is higher affinity than naturally occurring TCRs^21^. Comparisons of responses to CAR versus TCR ligation have demonstrated that CARs are inefficient at recruiting proximal signaling molecules to the immune synapse, rendering them unable to recognize antigen-low targets^13,14,22^. Thus, simplified CARs appear poorly matched for the complex and evolved T cell signaling machinery.

There is a pressing clinical need for CARs capable of recognizing antigen-low targets, as this mechanism of resistance has emerged for individuals treated with CD22, BCMA and CD19 CARs^9–12,56^. Given that CARs are deficient in recruiting and phosphorylating proximal signaling molecules, we set out to use these proteins to engineer an effective solution. Overexpressing the cytosolic adapter molecule SLP-76 enhanced CAR T cell responses to antigen-high target cells but was ineffective at enabling recognition of antigen-low targets. Only by tethering SLP-76 to the membrane did we enable CAR efficacy against antigen-low tumor cells both in vitro and in vivo. In its native form, SLP-76 is recruited to the membrane only after phosphorylation by ZAP-70, where it joins with LAT to make a scaffold for downstream effector functions. Although the recruitment and phosphorylation of ZAP-70 is a limiting factor in the CAR response, MT-SLP-76 circumvents this problem by delivering SLP-76 directly to the membrane where it is available for downstream functions. Researchers have termed LAT the bottleneck of TCR signaling^61^ and proposed that the amount of LAT determines T cell signaling strength^62,63^. Our data indicate that the bottleneck for CAR T cell signaling may be SLP-76, which has been previously shown to be capable of propagating a signal in the absence of LAT as long as it is translocated to the membrane^49^.

Overexpression of MT-SLP-76 rescues the performance of CARs against antigen-low tumors in clinically relevant animal models of low antigen density (CD19, CD22 and BCMA). This approach can be immediately deployed in the clinic alongside any CAR construct to overcome a frequently identified mechanism of antigen escape. This will have to be balanced against a theoretical risk of increased cytokine release syndrome and other toxicities related to CAR T cell expansion, such as immune effector cell-associated neurotoxicity and hemophagocytic lymphohistiocytosis-like syndrome. MT-SLP-76 can narrow the therapeutic window for targets shared with vital, normal tissues and may be best suited for antigens in hematologic malignancies or clean oncofetal antigens expressed on certain solid tumors^25,64–66^. MT-SLP-76 could also increase the potential for TCR-driven autoimmunity, although this could be mitigated by knocking out the endogenous TCR in clinical products. Although our studies in immunodeficient models demonstrate robust antitumor activity, the absence of a fully functional immune system limits our ability to fully elucidate cytokine-driven interactions, such as IL-2 effects on regulatory T cells, which could modulate antitumor responses. Future work should explore the effects of MT-SLP-76 in immunocompetent models.

MT-SLP-76 drives enhanced CAR T cell expansion in vivo, even in response to antigen-low targets. Importantly, although MT-SLP-76 CAR T cells expand to a greater extent, they ultimately contract similar to CAR-only controls, reducing concern for unchecked T cell proliferation. Although MT-SLP-76 increases the magnitude of the T cell response, this does not result in a loss of CAR T cell persistence. It has been surmised that the amplitude of signal strength in CD28-based CARs is responsible for their lack of persistence in human studies^41,67,68^, but our work indicates that factors other than high signal strength may drive CAR T cell deletion, a subject necessitating further study.

Multiple approaches for linking scFv-based recognition with the native TCR signaling chassis have been described, including STAR, HIT and TRUC receptors^16,26–28^. Although these methods can improve recognition of antigen-low targets, they also have limitations, including a lack of embedded co-stimulation, potential TCR chain mispairing and/or the need for gene knockout/knock-in technology. By contrast, MT-SLP-76 can be expressed alongside any CAR structure to enhance its signaling and capacity for antigen-low target recognition. In a model of CD22-low antigen density, MT-SLP-76 significantly outperformed some other systems that use proximal signaling molecules to enhance CAR T cell efficacy^14,53–55^.

In summary, we have drawn on insights from CAR T cell signaling biology to generate an effective and clinically deployable system for increasing CAR reactivity to antigen-low targets. This work adds to the growing literature demonstrating that the T cell’s internal signaling machinery can be co-opted and engineered to enhance CAR T cell activity and potentially improve clinical outcomes.

## Methods

All experiments were conducted in accordance with relevant ethical guidelines. The approving institutions and ethics committees are listed in the pertinent sections of the manuscript. Further information on research design is available in the Nature Research Reporting Summary linked to this article.

### Viral vector design

Retroviral vectors for CD19-4-1BBζ (FMC63), CD19-CD28ζ (FMC63), CD22-4-1BBζ (m971), HER2-4-1BBζ (4D5), ROR1-4-1BBζ (R11) and the lentiviral NFAT–GFP reporter vector have been previously described^13,69–72^. The sequence for the BCMA-4-1BBζ CAR was kindly provided by S. Riddell (Fred Hutchinson Cancer Center) and cloned into the MSGV1 retroviral vector^70^. CARs used a CD28 H/TM domain except CD22 (CD8 H/TM), BCMA (IgG4^S10P^ hinge and CD28 transmembrane) and ROR1 (IgG4^4/2NQ^ long spacer and CD28 transmembrane). CD19-4-1BBζ experiments used a CD28 H/TM except where appropriate to capture failure of the CD8 H/TM at low antigen density (Figs. 1c, 2d and 6b and Extended Data Fig. 2b). For the in vivo experiment described in Fig. 3g, the CAR construct was cloned in frame with truncated NGFR via a P2A polyprotein cleavage sequence to allow efficient gating of the CAR T cell population. For LCK, ZAP-70, LAT, SLP-76 and PLCγ1 overexpression, codon-optimized gBlocks (Integrated DNA Technologies) encoding the full-length proteins were cloned by In-Fusion (Takara Bio) in MSGV1 retroviral vectors. For detection purposes, a 2×HA tag was added at the C terminus of the LCK sequence. ZAP-70, LAT, SLP-76 and PLCγ1 were cloned in frame with truncated NGFR via a P2A sequence. To generate the membrane-tethered SLP-76 (MT-SLP-76) molecule, the N terminus of SLP-76 was linked to the CD8α H/TM and a VSV-G extracellular tag. A construct containing the VSV-G extracellular tag and the CD8α H/TM, lacking any intracellular signaling domain, was used as a control for co-transduction where indicated. MT-SLP-76 mutations and deletions were generated by PCR and In-Fusion cloning. The LAT–SLP-76 chimeric protein, including amino acids 1–35 of LAT and full-length SLP-76, as previously described, was produced by PCR and In-Fusion cloning into a vector including truncated NGFR after a P2A sequence^49^. The various CD22 CAR designs were generated via In-Fusion cloning of PCR products or gBlocks. The CD22-4-1BBζ-GRB2-SH2 construct includes a linker followed by the GRB2-SH2 at the C terminus of CD3ζ, as previously described^14^. The CD22-4-1BB-εRK-ζ CAR was generated by adding the CD3ε RK domain between the 4-1BB and CD3ζ sequences. The CD22-4-1BB-εICD-ζ CAR was generated by adding the CD3ε intracellular domain (including the RK motif) between the 4-1BB and CD3ζ sequences, as previously described^55^. The CD22-εBRS-4-1BBζ CAR was generated by adding the CD3ε BRS domain after the transmembrane domain, as previously described^54^. The CD22-4-1BB-εPRS-ITAM-ζ CAR includes the CD3ε PRS and ITAM components immediately before CD3ζ, as previously described^14^. Amino acid sequences of the constructs are provided in Supplementary Table 2.

### Viral vector production

Retroviral supernatants were generated by transiently transfecting 293GP cells as previously described^69^. Briefly, 6 × 10^6^–7 × 10^6^ 293GP cells were plated on 100-mm poly-D-lysine-coated plates (Corning) in DMEM supplemented with 10% fetal bovine serum (FBS), 10 mM HEPES and 1× penicillin–streptomycin–glutamine solution (Gibco). After 24 h, the cells were transfected with 4.5 μg of RD114, 9 μg of the plasmid encoding the gene of interest, 60 μl of Lipofectamine 2000 (Invitrogen) and 3 ml of Opti-MEM (Gibco). The medium was changed 24 h after transfection. The supernatant was collected 48 and 72 h after transfection and stored at –80 °C until use. Similarly, lentiviral supernatants for the NFAT–GFP reporter were produced by transient transfection of 293T cells using 10 μg of vector plasmid, along with 9 μg each of REV and GAG/Pol and 3.5 μg of VSV-G helper plasmids, as previously described^72^.

### Peripheral blood mononuclear cell and T cell isolation

Buffy coats, leukopaks or leukocyte reduction system chambers were obtained from consenting healthy donors through the Stanford Blood Center under an institutional review board-exempt protocol. Peripheral blood mononuclear cells were isolated using Ficoll-Paque Plus (GE Healthcare, 17–1440) density gradient centrifugation, according to the manufacturer’s instructions. In some experiments, T cells were isolated using RosetteSep Human T Cell Enrichment Cocktail (Stem Cell Technologies), according to the manufacturer’s protocol. Cells were cryopreserved using CryoStor CS10 freeze medium (Sigma-Aldrich).

### CAR T cell production

CAR T cells were generated as previously described^72^. Briefly, at day 0, peripheral blood mononuclear cells or isolated T cells were thawed and activated with Human T-Activator αCD3/CD28 Dynabeads (Gibco) at a 3:1 bead:cell ratio in complete AIM-V medium (Gibco; supplemented with 5% FBS, 10 mM HEPES, 1× penicillin–streptomycin–glutamine solution (Gibco) and 100 U ml^−1^ recombinant human IL-2 (Peprotech)). Retroviral transduction was performed on days 3 and 4 after activation. Twelve-well non-tissue culture-treated plates were coated with RetroNectin (Takara Bio) and blocked with 2% bovine serum albumin before incubation and centrifugation with the retroviral supernatant for at least 2 h at 32 °C and 2,174*g*. Coexpression of CAR and proximal signaling molecules was achieved by simultaneous transduction with two separate viral vectors. The supernatant was removed, and 0.5 × 10^6^ T cells were added to each well in 1 ml of complete AIM-V medium.

For experiments using the NFAT-inducible GFP reporter, activated T cells were transduced on day 3 with the NFAT–GFP lentiviral supernatant and then on days 4 and 5 with the CAR ± MT-SLP-76 retroviral supernatants. Dynabeads were removed on day 5 or 6 after activation by magnetic separation, and the T cells were maintained in culture in complete AIM-V at a density of 0.3 × 10^6^ cells per ml until days 10–14, with medium changes performed every 2–3 days.

### Cell lines

All cell lines were cultured in complete RPMI-1640 medium (Gibco) supplemented with 10% FBS, 10 mM HEPES and 1× penicillin–streptomycin–glutamine solution (Gibco) and tested negative for mycoplasma using a MycoAlert Mycoplasma Detection kit (Lonza). The Nalm6-GFP Luciferase B-ALL cell line was obtained from S. Grupp (University of Pennsylvania). The generation of Nalm6 cell lines with variable levels of CD19, CD22, HER2 and ROR1 expression was previously described^10,13,71^. The OPM-2-GFP-luciferase multiple myeloma cell line was obtained from E. Smith (Dana-Farber Cancer Institute). The Nalm6-CD22^1,300^ cell line^10^ was further engineered using CRISPR–Cas9 to remove β_2_-microglobulin to eliminate the graft versus leukemia effect. The following single guide RNA (sgRNA) target sequences were used: 5′-ACTCACGCTGGATAGCCTCC-3′^73^ (*B2M* sgRNA 1) and 5′-GAGTAGCGCGAGCACAGCTA-3′^74^ (B2M sgRNA 2). Guide RNAs were purchased from Synthego, and Alt-R S.p. Cas9 Nuclease V3 (10 μg μl^−1^) and Nuclease-Free Duplex Buffer were purchased from IDT. Editing was performed as previously described^31^, and *B2M-*KO cells were identified as HLA-A2 negative (APC, clone BB7.2, BD) and flow sorted.

### Flow cytometry

Surface and intracellular markers were evaluated using a BD Fortessa or an Agilent NovoCyte Quanteon or Penteon flow cytometer and FlowJo software (BD) for data analysis. CD19 CAR was detected using an idiotype antibody specific for the FMC63 scFv (1:400). HER2, CD22, BCMA and ROR1 CARs were detected using the respective target recombinant protein (R&D Systems, 1:400). Idiotype antibodies and recombinant proteins were fluorophore conjugated using DyLight 650 or DyLight 488 Microscale Antibody Labeling kits (Invitrogen) following the manufacturer’s instructions. The surface detection of overexpressed proximal signaling molecules was evaluated via VSV-G (FITC or biotin, polyclonal, Abcam, 1:100), NGFR (BV421, clone C40-1457, BD Biosciences, 1:200), HA (Pacific Blue, clone 16B12, BioLegend, 1:100) and streptavidin (PE, BioLegend, 1:100) staining.

T cells were further assessed for the expression of CD4 (BUV 395, clone SK3, BD Biosciences, 1:100), CD8 (BUV 805, clone SK1, BD Biosciences, 1:200), CD45 (PerCP–Cy5.5, clone HI30, Invitrogen), CD62L (BV605, clone DREG-56, BD Biosciences, 1:100) and CD45RA (BV711, clone HI100, BD Biosciences, 1:100). Intracellular staining of proximal T cell signaling molecules was performed according to the manufacturer’s protocol using a Foxp3/Transcription Factor Staining Buffer Set (eBioscience). The following antibodies were used for intracellular staining: LCK (Alexa Fluor 647, clone Lck-01, BioLegend, 1:200), LAT (Alexa Fluor 647, clone 661002, R&D Systems, 1:200), PLCγ1 (Alexa Fluor 647, clone 10, BD Biosciences, 1:12.5), ZAP-70 (Alexa Fluor 647, clone A16043B, BioLegend, 1:100) and SLP-76 (Alexa Fluor 647, clone H3, BD Biosciences, 1:12.5).

Tumor cells were assessed for target antigen expression with antibodies recognizing CD19 (APC or PE, clone HIB19, BioLegend, 1:50), CD22 (APC, clone HIB22, BioLegend, PE, clone S-HCL-1, BioLegend, 1:50), HER2 (PE–Cy7 or PE, clone 24D2, BioLegend, 1:50), ROR1 (PE–Cy7, clone 2A2, BioLegend, 1:50) and BCMA (PE, clone 19F2, BioLegend, 1:100). A fixable viability dye (eFluor 780, eBioscience, 1×) was used in all in vivo and intracellular flow cytometry analyses. CD19, CD22, HER2 and BCMA antigen density was estimated using BD QuantiBRITE PE beads, as per the manufacturer’s protocol. Mean density (±s.d.) calculated in two independent quantification experiments is reported in Supplementary Table 1.

### Mass spectrometry

CD19-CD8H/TM-4-1BBζ and CD19-CD28H/TM-4-1BBζ T cells (5 × 10^6^) were stimulated with 5 μg ml^−1^ anti-CD19 CAR idiotype and a goat anti-mouse cross-linking antibody (Jackson ImmunoResearch) and incubated at 37 °C for 5, 15 or 90 min. After stimulation, cells were quenched with cold PBS, and cell pellets were collected and flash frozen. Samples were then dissolved in 100 µl of lysis buffer (0.5 M triethylammonium bicarbonate and 0.05% sodium deoxycholate), subjected to tip sonication (Q700, QSonica, amplitude = 10, pulses of 2 s on/2 s off, 20 s total processing time per sample, on ice) and centrifuged at 17,000*g* at 4 °C for 10 min. Protein concentration was measured using a Pierce Bradford Protein Assay kit (Thermo Fisher Scientific), per the manufacturer’s instructions. Equal protein amounts (100 µg per sample) were adjusted to a uniform volume with lysis buffer. Samples were reduced with 4 µl of reducing reagent (Sigma) at 60 °C for 1 h and alkylated with 2 µl of alkylating reagent (Sigma) at room temperature for 15 min. Digestion was performed with 4 µg of trypsin/Lys-C (Promega) overnight at room temperature in the dark.

TMTpro reagents (Thermo Fisher Scientific) were reconstituted in 20 µl of anhydrous acetonitrile (Sigma) and added to each sample, followed by incubation at room temperature for 1 h. Labeling was quenched with 8 µl of 5% hydroxylamine for 15 min. Samples were combined and dried using a SpeedVac (Eppendorf 5301). Phosphopeptides were sequentially enriched using High-Select TiO_2_ and Fe-NTA phosphopeptide enrichment kits (Thermo Fisher Scientific), according to the manufacturer’s instructions. Enriched fractions were reconstituted in 0.1% formic acid and analyzed by LC–MS (nano-easy LC 1200, Thermo Q Exactive).

Raw LC–MS data were processed using Proteome Discoverer 2.3 (Thermo Fisher Scientific) with a target-decoy search via Byonic against the *Homo sapiens* SwissProt database (TaxID 9606, v2019-12-30). Search parameters included up to two missed cleavages, 20-ppm precursor mass tolerance, a minimum peptide length of six and dynamic modifications of oxidation (M; rare 1), deamidation (N, Q; rare 1) and phosphorylation (S, T, Y; common 2). Methylthio (C) and TMTpro (K, peptide N terminus) were set as static modifications. Peptide level confidence was set at *q* < 0.01 (<1% false discovery rate). Counts per million normalization mode of total peptide amount was applied per sample, and downstream analyses, including PC analysis and differential expression, was performed on the log_2-_transformed counts matrix. Differential peptide analyses were conducted using an empirical Bayes moderated *t*-test^75^.

### Cytokine production assays

At days 10–14 after activation, CAR T cells were cocultured with tumor cells at a 1:1 effector:target (E:T) ratio in complete RPMI medium. In some experiments, a plate was coated with recombinant CD22 protein at various concentrations and used for stimulation. After 24 h, supernatants were collected, and IL-2 or IFN-γ was measured by enzyme-linked immunosorbent assay following the manufacturer’s protocol (BioLegend).

### Cytotoxicity assays

At days 10–14 after activation, CAR T cells were cocultured with various GFP^+^ tumor cells in a 1:1 E:T ratio in complete RPMI medium for 72 h. Cocultures were imaged every 3 h with an Incucyte S3 Live-Cell Analysis System (Sartorius). Target cell killing was quantified by measuring the total green fluorescence intensity over time using the basic analyzer feature on the Incucyte S3 software. The reported cytotoxicity index was calculated by dividing the total green fluorescence intensity at each time point by the measurement at time 0.

### Luminex assay

CD19-4-1BBζ ± MT-SLP-76 CAR T cells were cocultured with Nalm6-CD19^600^ or Nalm6-CD19^20,100^ cells for 24 h, and the supernatant was collected and frozen. The assay was performed by the Human Immune Monitoring Center Immunoassay Team at Stanford University. Kits were purchased from EMD Millipore (Human 80 Plex kit) and included three panels. Panel 1 was Milliplex HCYTA-60K-PX48, panel 2 was Milliplex HCP2MAG-62K-PX23, and panel 3 included the Milliplex HSP1MAG-63K-06 and HADCYMAG-61K-03 (Resistin, Leptin and HGF) to generate a nine-plex. The assay setup followed the recommended protocol. Samples were diluted threefold for panels 1 and 2 and tenfold for panel 3 and incubated overnight at 4 °C with antibody-linked magnetic beads in a 96-well plate (25 µl per well) on an orbital shaker (500–600 rpm). Plates were washed twice using a Biotek ELx405 washer (BioTek Instruments), followed by a 1-h incubation with biotinylated detection antibody and a 30-min incubation with streptavidin–PE, both at room temperature with shaking. After a final wash, PBS was added, and samples were read on a Luminex FlexMap3D instrument (≥50 beads per cytokine per sample). Custom Assay Chex control beads (Radix Biosolutions) were included in all wells. All wells met quality control metrics (bead count of >50). Data are shown as heat maps of log_2_ (FC) from unstimulated controls. All samples were run in technical duplicate.

### NFAT–GFP T cell activation assay

CD19-4-1BBζ CAR T cells co-transduced with either MT-SLP-76 variants or the VSV-G-CD8H/TM control and an NFAT–GFP reporter^72^ were stimulated with 1 or 2.5 μg ml^−1^ of idiotype antibody to CD19 CAR and a goat anti-mouse cross-linking antibody and incubated at 37 °C for 6 h. In parallel, as a positive control for maximal NFAT activation, the same CAR T cells were stimulated with Cell Stimulation Cocktail (500×, eBioscience) containing phorbol 12-myristate 13-acetate and ionomycin. Following stimulation, cells were washed with cold PBS containing 2% FBS, stained for CAR, VSV-G tag, CD4 and CD8 and analyzed by flow cytometry. NFAT activation was quantified as the gMFI of GFP⁺ cells within the CAR⁺, VSV-G⁺ population.

### In vivo xenograft models

Animal studies were performed according to a Stanford Institutional Animal Care and Use Committee-approved protocol (protocol 33698). Immunodeficient NOD.Cg-*Prkdc*^*scid*^*Il2rg*^tm1Wjl^/SzJl mice were purchased from The Jackson Laboratory or were bred in-house. Five- to 10-week-old male or female mice were used for all experiments. Mice were housed at 22 °C with 50% humidity under a 12-h light/12-h dark cycle. In the CD19-low model, mice were injected with 1 × 10^6^ Nalm6-CD19^600^ cells and treated with 3 × 10^6^ CD19-4-1BBζ CAR T cells ± MT-SLP-76 or an equivalent number of untransduced control T cells (mock) 4 days later, following a previously described dosing scheme^13^. In the CD22-low model, mice were injected with 1 × 10^6^ Nalm6-CD22^1,300^ cells and treated with 5 × 10^6^–7 × 10^6^ CD22-4-1BBζ CAR T cells ± MT-SLP-76 or an equivalent number of control T cells (mock or MT-SLP-76-only transduced cells) 3 days later. T cell dose was determined based on tumor engraftment before treatment (5 × 10^6^ cells were administered when total flux values were below 1.5 × 10⁷ p s^−1^ and 6 × 10^6^–7 × 10^6^ cells for higher values). For the CD19 stress test model, mice were injected with 1 × 10^6^ wild-type Nalm6-CD19^20,100^ cells and treated with 1 × 10^6^ CD19-4-1BBζ CAR T cells ± MT-SLP-76 or an equivalent number of control T cells (MT-SLP-76 only) after 3 days. In the persistence experiments, mice were injected with 1 × 10^6^ Nalm6-CD19^20,100^ cells and treated with 5 × 10^6^ CD19-CD28ζ or 7 × 10^6^ CD19-4-1BBζ CAR T cells ± MT-SLP-76 or an equivalent number of mock cells 3 days later. T cell doses were chosen to ensure tumor clearance, accounting for differences in potency between co-stimulatory domains. For the multiple myeloma model, mice were engrafted with 1 × 10^6^ OPM-2 cells and treated with 0.4 × 10^6^ BCMA-4-1BBζ CAR T cells ± MT-SLP-76 or an equivalent number of control mock cells after 3 weeks. In the OTOTT model, mice were injected with 1 × 10^6^ ROR1^+^-Nalm6 cells and treated with 5 × 10^6^ ROR1-4-1BBζ CAR T cells ± MT-SLP-76 or an equivalent number of control T cells (MT-SLP-76 only) 3 days later. Mice were weighed daily and were humanely killed if their weight dropped by 20% from baseline or if they displayed significant signs of distress. Disease progression was monitored at least weekly via BLI. Mice were injected intraperitoneally with 200 μl of 15 mg ml^−1^ D-luciferin and imaged with an IVIS imaging system (PerkinElmer) or an Ami HTX (Spectral Instruments Imaging) 4 min later, with an exposure time of 30 s. Saturated images were reacquired using autoexposure. Regions of interest of consistent shape were drawn around each mouse, and total fluxes were calculated using LivingImage software (PerkinElmer) and Aura software (Spectral Instruments Imaging). Mice were humanely killed when they showed signs of morbidity or hind leg paralysis or developed solid masses, in compliance with the approved ethical protocol. No statistical methods were used to predetermine sample sizes. Group sizes were informed by previously published and validated models^13,31^. Mice were randomized before treatment to equalize tumor burden. No animals or data points were excluded. Injections were performed by a blinded technician.

### Single-cell RNA sequencing

CD22-4-1BBζ T ± MT-SLP-76 cells were cocultured with or without Nalm6-CD22^1,300^ cells for 5 and 24 h at a 1:1 E:T ratio. Cells were then stained with Viability Dye and anti-CD4, anti-CD8 and recombinant CD22 protein for CAR detection. CAR^+^ T cells were gated as live, GFP^−^, CD4^+^, CD8^+^ and CAR^+^ before isolation using a BD FACS Aria cell sorter. Approximately 60,000 live cells per condition were collected for each T cell donor (two donors total), which were then pooled in each condition before library preparation using the 10× Chromium Controller and a Chromium Single Cell 5′ Library Construction kit. Suspended cells were loaded onto the Chromium controller to generate single-cell Gel Bead-In-Emulsions. cDNA libraries were generated by reverse transcription and sample indexing using a C1000 Touch Thermal cycler with the 96-Deep Well Reaction Module (Bio-Rad). Fragmenting, poly(A) tailing, adaptor ligation and PCR amplification with sample index primers were used for multiplexing libraries. The final products were quantified using a Bioanalyzer 2100 system (Agilent). The 10× scRNA-seq libraries were sequenced as recommended by the manufacturer on a Nova-seq 6000 S4 Flow Cell at approximately 25,000 reads per cell. Raw sequencing reads were demultiplexed using CellRanger mkfastq and aligned to the human reference transcriptome using the CellRanger count v.6.0 pipeline with all default parameters. Donors were demultiplexed using mitochondrial DNA genotypes and the mgatk v.0.6.2 software^76^. Downstream analyses, including cell filtering, cell-type identification, module score estimation and reduced dimensionality visualizations, were conducted using Seurat v.5.1 (ref. ^77^). Notably, to identify single-cell RNA-sequencing clusters, count data were first log normalized, and the top 2,000 most variable genes were selected for scaling and dimensionality reduction via PC analysis. The first 30 PCs were used to construct a nearest neighbor graph where clusters were identified via Louvain clustering with the FindClusters defined at 2. The same PCs were then used to embed cells via the UMAP algorithm. For cell-type assignment, a cell was classified as CD8^+^ if it was in a cluster where the total number of reads mapped to CD8A and CD8B was more than twice the number of reads mapped to CD4; otherwise the cell cluster was classified as CD4^+^. Pseudobulk samples were created by aggregating read counts from all cells from either donor either in the unstimulated condition or the stimulated condition for specific cell types. Per-cell module scores were computed using the FindModuleScores with default parameters for previously described gene sets^31^.

### Statistical analysis

Data were visualized and analyzed using Excel v.16.83 (Microsoft), GraphPad Prism v.10.2.2 (GraphPad) or R v.4.2.2 (R Core Team) software. Graphs represent either individual values or group mean values ± s.d. for in vitro experiments and group mean values ± s.e.m. for in vivo experiments. The statistical analyses performed are specified in the individual figure legends. Data distribution was assumed to be normal, but this was not formally tested. *P* values of less than 0.05 were considered statistically significant. All genomics and proteomics analyses were performed using the R analysis software environments noted above.

### Reporting summary

Further information on research design is available in the Nature Portfolio Reporting Summary linked to this article.

## Supplementary information

Supplementary Information Supplementary Tables 1 and 2 and Supplementary Figs. 1 and 2.

Reporting Summary

## Source data

Source Data Figs. 1–6 Statistical source data.

Source Data Extended Data Figs. 2, 3 and 5 Statistical source data.

## Data Availability

All mass spectrometry data have been deposited to the ProteomeXchange Consortium via the PRIDE partner repository (dataset identifier PXD053205; https://www.ebi.ac.uk/pride/archive/projects/PXD053205). The scRNA-seq datasets have been deposited in the NCBI Gene Expression Omnibus (GEO) and are accessible through the GEO series accession number GSE270399. The remaining data are available within the article and the Supplementary Information. Source data are provided with this paper.
