# Supplementary material for: Engineering T cells with a membrane-tethered version of SLP-76 overcomes antigen-low resistance to CAR T cell therapy
Source: Nat Cancer. 2025 Oct 23;6(12):1940–54. doi: 10.1038/s43018-025-01056-4 (PMC12727510; doi:10.1038/s43018-025-01056-4)
Supplement: Supplementary file 1 — Supplementary Tables 1 and 2 and Supplementary Figs. 1 and 2. [file 43018_2025_1056_MOESM1_ESM.pdf]

nature cancer

Supplementary information https://doi.org/10.1038/s43018-025-01056-4

Engineering T cells with a membrane-tethered version of SLP-76 overcomes antigen-low resistance to CAR T cell therapy

In the format provided by the authors and unedited

Supplementary Table 1: Antigen density measurements in cell lines.

Cell line	Number of molecules/cell
Nalm6-CD19^{20,100} (wildtype)	20,068 ± 108
Nalm6-CD19^{600}	577 ± 170
Nalm6-CD19^{2,500}	2,471 ± 303
Nalm6-CD19^{2,900}	2,943 ± 237
Nalm6-CD19^{21,500}	21,471 ± 259
Nalm6-CD19^{25,900}	25,923 ± 2,062
Nalm6-CD19^{59,300}	59,287 ± 4,852
Nalm6-CD19^{249,700}	249,718 ± 10,136
Nalm6-CD22^{1,300}	1,310 ± 84
Nalm6-CD22^{2,600} (wildtype)	2596 ± 21
Nalm6-HER2^{1,700}	1,693 ± 41
Nalm6-HER2^{10,600}	10,575 ± 231
Nalm6-HER2^{105,100}	105,110 ± 2,848
OPM-2 (BCMA)	1,206 ± 168

Supplementary Table 2: Amino acid sequences of the constructs used in this study.

**scFvs (with 5' leader sequence and linker between VH and VL chains)**	
CD19 (FMC63)	MLLLVTSLLLCELPHPAFLLIPDIQMTQTTSSLSASLGDRVTISCRASQDISKYLNWYQQKPDGTVKLLIYHTSRLHSGVPSRFSGSGSGTDYSLTISNLEQEDIATYFCQQGNTLPYTFGGGTKLEITGSTSGSGKPGSGEGSTKGEVKLQESGPGLVAPSQSLSVTCTVSGVSLPDYGVSWIRQPPRKGLEWLGVIWGSETTYYNSALKSRLTIIKDNSKSQVFLKMNSLQTDDTAIYYCAKHYYYGGSYAMDYWGQGTSVTVSS
CD22 (m971)	MLLLVTSLLLCELPHPAFLLIPQVQLQQSGPGLVKPSQTLSLTCAISGDSVSSNSAAWNWIRQSPSRGLEWLGRTYYRSKWYNDYAVSVKSRITINPDTSKNQFSLQLNSVTPEDTAVYYCAREVTGDLEDAFDIWGQGTMVTVSSGGGGSDIQMTQSPSSLSASVGDRVTITCRASQTIWSYLNWYQQRPGKAPNLLIYAASSLQSGVPSRFSGRGSGTDFTLTISSLQAEDFATYYCQQSYSIPQTFGQGTKLEIKAA
HER2 (4D5)	MARSVTLVFLVLVSLTGLYAADIQMTQSPSSLSASVGDRVTITCRASQDVNTAVAWYQQKPGKAPKLLIYSASFLESGVPSRFSGSRSGTDFTLTISSLQPEDFATYYCQQHYTTPPTFGQGTKVEIKGSTSGSGKPGSGEGSGEVQLVESGGGLVQPGGSLRLSCAASGFNIKDTYIHWVRQAPGKGLEWVARIYPTNGYTRYADSVKGRFTISADTSKNTAYLQMNSLRAEDTAVYYCSRWGGDGFYAMDVWGQGTLVTVSS
ROR1 (R11)	MLLLVTSLLLCELPHPAFLLIPQSVKESEGDLVTPAGNLTLTCTASGSDINDYPISWVRQAPGKGLEWIGFINSGGSTWYASWVKGRFTISRTSTTVDLKMTSLTTDDTATYFCARGYSTYYGDFNIWGPGTLVTISSGGGGSGGGGSGGGGSELVMTQTPSSTSGAVGGTVTINCQASQSIDSNLAWFQQKPGQPPTLLIYRASNLASGVPSRFSGSRSGTEYTLTISGVQREDAATYYCLGGVGNVSYRTSFGGGTEVVVK

**Hinge-Transmembrane Domains**	
CD28 H/TM	AAAIEVMYPPPYLDNEKSNGTIIHVKGKHLCPSPLFPGPSKPFWVLVVVGGVLACYSLLVTVAFIIFWV
CD8 H/TM	AAATTTPAPRPPTPAPTIASQPLSLRPEACRPAAGGAVHTRGLDFACDIYIWAPLAGTCGVLLLSLVITLYC

**Intracellular Signaling Domains**	
CD3ζ	RVKFSRSADAPAYKQGQNQLYNELNLGRREEYDVLDKRRGRDPEMGGKPRRKNPQEGLYNELQKDKMAEAYSEIGMKGERRRGKGHDGLYQGLSTATKDTYDALHMQALPPR
Lck	MGCGCSSHPEDDWMENIDVCENCHYPIVPLDGKGTLLIRNGSEVRDPLVTYEGSNPPASPLQDNLVIALHSYEPSHDGDLGFEKGEQLRILEQSGEWWKAQSLTTGQEGFIPFNFVAKANSLEPEPWFFKNLSRKDAERQLLAPGNTHGSFLIRESESTAGSFSLSVRDFDQNQGEVVKHYKIRNLDNGGFYISPRITFPGLHELVRHYTNASDGLCTRLSRPCQTQKPQKPWWEDEWEVPRETLKLVERLGAGQFGEVWMGYYNGHTKVAVKSLKQGSMSPDAFLAEANLMKQLQHQRLVRLYAVVTQEPIYIITEYMENGSLVDFLKTPSGIKLTINKLLDMAAQIAEGMAFIEERNYIHRD

	LRAANILVSDTLSCKIADFGLARLIEDNEYTAREGAKFPIKWTAPEA INYGTFTIKSDVWSFGILLTEIVTHGRIPYPGMTNPEVIQNLERGYR MVRPDNCPEELYQLMRLCWKERPEDRPTFDYLRSVLEDFFTATEG QYQPQP
ZAP-70	MPDPAAHLPFFYGSISRAEAEEHLKLAGMADGLFLLRQCLRSLGG YVLSLVHDVRFHHFPIERQLNGTYAIAGGKAHCGPAELCEFYSRDP DGLPCNLRKPCNRPSGLEPQPGVFDCLRDAMVRDYVRQTWKLEG EALEQAIISQAPQVEKLIATTAHERMPWYHSSLTREEAERKLYSGA QTDGKFLLRPRKEQGTYALSLIYGKTVYHYLISQDKAGKYCIPEGT KFDTLWQLVEYLKLKADGLIYCLKEACPNSSASNASGAAAPTLPA HPSTLTHPQRRIDTLNSDGYTPEPARITSPDKPRPMPMDTSVYESPY SDPEELKDKKLFLKRDNLLIADIELGCGNFGSVRQGVYRMRKKQID VAIKVLKQGTEKADTEEMMREAQIMHQLDNPYIVRLIGVCQAEAL MLVMEMAGGGPLHKFLVGKREEIPVSNVAELLHQVSMGMKYLEE KNFVHRDLAARNVLLVNRHYAKISDFGLSKALGADDSYYTARSA GKWPLKWYAPECINFRKFSSRSDVWSYGVTMWEALSYGQKPYKK MKGPEVMAFIEQGKRMECPPECPPELYALMSDCWIYKWEDRPDFL TVEQRMRACYYSLASKVEGPPGSTQKAEAACA
LAT	MEEAILVPCVLGLLLLPILAMLMALCVHCHRLPGSYDSTSSDSLYP RGIQFKRPHTVAPWPPAYPPVTSYPPLSQPDLLPIPRSPQPLGGSHRT PSSRRDSDGANSVASYENEGASGIRGAQAGWGVWGPSWTRLTPVS LPPEPACEDADEDEDDYHNPGYLVVLPDSTPATSTAAPSAPALSTP GIRDSAFSMESIDDYVNVPESGESAEASLDGSREYVNVSQELHPGA AKTEPAALSSQEAEEVEEEGAPDYENLQELN
SLP-76	MALRNVPFRSEVLGWDPDSLADYFKKLNYKDCEKAVKKYHIDGA RFLNLTENDIQKFPKLRVPILSKLSQEINKNEERRSIFTRKPQVPRFPE ETESHEEDNGGWSSFEEDDYESPNDDQDGEDDGDYESPNEEEEAP VEDDADYEPPPSNDEEALQNSILPAKPFPNSNSMYIDRPPSGKTPQQ PPVPPQRPMAALPPPPAGRNHSPLPPPQTNHEEPSRSRNHKTAKLPA PSIDRSTKPPLDRSLAPFDREPFTLGKKPPFSDKPSIPAGRSLGEHLP KIQKPPLPPTTERHERSSPLPGKKPPVPKHGWGPDRRENDEDDVHQ RPLPQPALLPMSSNTFPSRSTKPSPMNPLPSSHMPGAFSESNSSFPQS ASLPPYFSQGPSNRPPIRAEGRNFPLPLPNKPRPPSPAEEENSLNEEW YVSYITRPEAEAALRKINQDGTFLVRDSSKKTTTNPYVLMVLYKD KVYNIQIRYQKESQVYLLGTGLRGKEDFLSVSDIIDYFRKMPLLLID GKNRGSRYQCTLTHAAGYPGNS
SLP-76 with linker (as used in MT-SLP-76)	GSGGGGSALRNVPFRSEVLGWDPDSLADYFKKLNYKDCEKAVKK YHIDGARFLNLTENDIQKFPKLRVPILSKLSQEINKNEERRSIFTRKP QVPRFPEETESHEEDNGGWSSFEEDDYESPNDDQDGEDDGDYESP NEEEEAPVEDDADYEPPPSNDEEALQNSILPAKPFPNSNSMYIDRPP SGKTPQQPPVPPQRPMAALPPPPAGRNHSPLPPPQTNHEEPSRSRNH KTAKLPAPSIDRSTKPPLDRSLAPFDREPFTLGKKPPFSDKPSIPAGR SLGEHLPKIQKPPLPPTTERHERSSPLPGKKPPVPKHGWGPDRREND EDDVHQRPLPQPALLPMSSNTFPSRSTKPSPMNPLPSSHMPGAFSES NSSFPQSASLPPYFSQGPSNRPPIRAEGRNFPLPLPNKPRPPSPAEEEN SLNEEWYVSYITRPEAEAALRKINQDGTFLVRDSSKKTTTNPYVLM

	VLYKDKVYNIQIRYQKESQVYLLGTGLRGKEDFLSVSDIIDYFRKM PLLLIDGKNRGSRYQCTLTHAAGYPGNS
LAT-SLP-76 chimeric protein	MEEAILVPCVLGLLLLPILAMLMALCVHCHRLPGSMALRNVPFRSE VLGWDPDSLADYFKKLNYKDCEKAVKKYHIDGARFLNLTENDIQ KFPKLRVPILSKLSQEINKNEERRSIFTRKPQVPRFPEETESHEEDNG GWSSFEEDDYESPNDDQDGEDDGDYESPNEEEEAPVEDDADYEPP PSNDEEALQNSILPAKPFPNSNSMYIDRPPSGKTPQQPPVPPQRPMA ALPPPPAGRNHSPLPPPQTNHEEPSRSRNHKTAKLPAPSIDRSTKPPL DRSLAPFDREPFTLGKKPPFSDKPSIPAGRSLGEHLPKIQKPPLPPTT ERHERSSPLPGKKPPVPKHGWGPDRRENDEDDVHQRPLPQPALLP MSSNTFPSRSTKPSPMNPLPSSHMPGAFSESNSSFPQSASLPPYFSQG PSNRPPIRAEGRNFPLPLPNKPRPPSPAEEENSLNEEWYVSYITRPEA EAALRKINQDGTFLVRDSSKKTTTNPYVLMVLYKDKVYNIQIRYQ KESQVYLLGTGLRGKEDFLSVSDIIDYFRKMPLLLIDGKNRGSRYQ CTLTHAAGYP
PLCγ1	MAGAASPCANGCGPGAPSDAEVLHLCRSLEVGTVMTLFYSKKSQR PERKTFQVKLETRQITWSRGADKIEGAIDIREIKEIRPGKTSRDFDRY QEDPAFRPDQSHCFVILYGMEFRLKTLSLQATSEDEVNMWIKGLT WLMEDTLQAPTPLQIERWLRKQFYSVDRNREDRISAKDLKNMLSQ VNYRVPNMRFLRERLTDLEQRSGDITYGQFAQLYRSLMYSAQKTM DLPFLEASTLRAGERPELCRVSLPEFQQFLLDYQGELWAVDRLQVQ EFMLSFLRDPLREIEEPYFFLDEFVTFLFSKENSVWNSQLDAVCPDT MNNPLSHYWISSSHNTYLTGDQFSSESSLEAYARCLRMGCRCIELD CWDGPDGMPVIYHGHTLTTKIKFSDVLHTIKEHAFVASEYPVILSIE DHCSIAQQRNMAQYFKKVLGDTLLTKPVEISADGLPSPNQLKRKIL IKHKKLAEGSAYEEVPTSMMYSENDISNSIKNGILYLEDPVNHEWY PHYFVLTSSKIYYSEETSSDQGNEDEEEPKEVSSSTELHSNEKWFHG KLGAGRDGRHIAERLLTEYCIETGAPDGSFLVRESETFVGDYTLSF WRNGKVQHCRIHSRQDAGTPKFFLTDNLVFDSLYDLITHYQQVPL RCNEFEMRLSEPVPQTNAHESKEWYHASLTRAQAEHMLMRVPRD GAFLVRKRNEPNSYAISFRAEGKIKHCRVQQEGQTVMLGNSEFDSL VDLISYYEKHPLYRKMKLRYPINEEALEKIGTAEPDYGALYEGRNP GFYVEANPMPTFKCAVKALFDYKAQREDELTFIKSAIIQNVEKQEG GWWRGDYGGKKQLWFPSNYVEEMVNPVALEPEREHLDENSPLG DLLRGVLDVPACQIAIRPEGKNNRLFVFSISMASVAHWSLDVAADS QEELQDWVKKIREVAQTADARLTEGKIMERRKKIALELSELVVYC RPVPFDEEKIGTERACYRDMSSFPETKAEKYVNKAKGKKFLQYNR LQLSRIYPKGQRLDSSNYDPLPMWICGSQLVALNFQTPDKPMQMN QALFMTGRHCGYVLQPSTMRDEAFDPFDKSSLRGLEPCAISIEVLG ARHLPKNGRGIVCPFVEIEVAGAEYDSTKQKTEFVVDNGLNPVWP AKPFHFQISNPEFAFLRFVVYEEDMFSDQNFLAQATFPVKGLKTGY RAVPLKNNYSEDLELASLLIKIDIFPAKENGDLSPFSGTSLRERGSDA SGQLFHGRAREGSFESRYQQPFEDFRISQEHLADHFDSRERRAPRRT RVNGDNRL
CD28	RSKRSRLLHSDYMNMTPRRPGPTRKHYQPYAPPRDFAAYRS
4-1BB	KRGRKKLLYIFKQPFMRPVQTTQEEDGCSCRFPEEEEGGCEL

4-1BBζ-GRB2-SH2	KRGRKKLLYIFKQPFMRPVQTTQEEDGCSCRFPEEEEGGCELRVKF SRSADAPAYKQGQNQLYNELNLGRREEYDVLDKRRGRDPEMGGK PRRKNPQEGLYNELQKDKMAEAYSEIGMKGERRRGKGHDGLYQG LSTATKDTYDALHMQALPPRGSTSGSGKPGSGEGSTKGWFFGKIPR AKAEEMLSKQRHDGAFLIRESESAPGDFSLSVKFGNDVQHFKVLR DGAGKYFLWVVKFNSLNELVDYHRSTSVSRNQQIFLRDIE
4-1BB-εRK-ζ	KRGRKKLLYIFKQPFMRPVQTTQEEDGCSCRFPEEEEGGCELRKGQ RDLYRVKFSRSADAPAYKQGQNQLYNELNLGRREEYDVLDKRRG RDPEMGGKPRRKNPQEGLYNELQKDKMAEAYSEIGMKGERRRGK GHDGLYQGLSTATKDTYDALHMQALPPR
εBRS-4-1BBζ	KNRKAKAKKRGRKKLLYIFKQPFMRPVQTTQEEDGCSCRFPEEEE GGCELRVKFSRSADAPAYKQGQNQLYNELNLGRREEYDVLDKRR GRDPEMGGKPRRKNPQEGLYNELQKDKMAEAYSEIGMKGERRRG KGHDGLYQGLSTATKDTYDALHMQALPPR
4-1BB-εPRS-ITAM-ζ	KRGRKKLLYIFKQPFMRPVQTTQEEDGCSCRFPEEEEGGCELRPPP VPNPDYEPIRKGQRDLYSGLNQRRIRVKFSRSADAPAYKQGQNQL YNELNLGRREEYDVLDKRRGRDPEMGGKPRRKNPQEGLYNELQK DKMAEAYSEIGMKGERRRGKGHDGLYQGLSTATKDTYDALHMQ ALPPR
4-1BB-εICD-ζ	KRGRKKLLYIFKQPFMRPVQTTQEEDGCSCRFPEEEEGGCELKNRK AKAKPVTRGAGAGGRQRGQNKERPPPVPNPDYEPIRKGQRDLYSG LNQRRIRVKFSRSADAPAYKQGQNQLYNELNLGRREEYDVLDKRR GRDPEMGGKPRRKNPQEGLYNELQKDKMAEAYSEIGMKGERRRG KGHDGLYQGLSTATKDTYDALHMQALPPR

**Tags**	
VSV-G	YTDIEMNRLGK
2xHA	YPYDVPDYAYPYDVPDYA
tNGFR	MGAGATGRAMDGPRLLLLLLLGVSLGGAKEACPTGLYTHSGECC KACNLGEGVAQPCGANQTVCEPCLDSVTFSDVVSATEPCKPCTEC VGLQSMSAPCVEADDAVCRCAYGYYQDETTGRCEACRVCEAGSG LVFSCQDKQNTVCEECPDGTYSDEANHVDPCLPCTVCEDTERQLR ECTRWADAECEEIPGRWITRSTPPEGSDSTAPSTQEPEAPPEQDLIAS TVAGVVTTVMGSSQPVVTRGTTDNLIPVYCSILAAVVVGLVAYIAF KR

**P2A**	
P2A with Furin cleavage site	RKRRGSGATNFSLLKQAGDVEENPGP

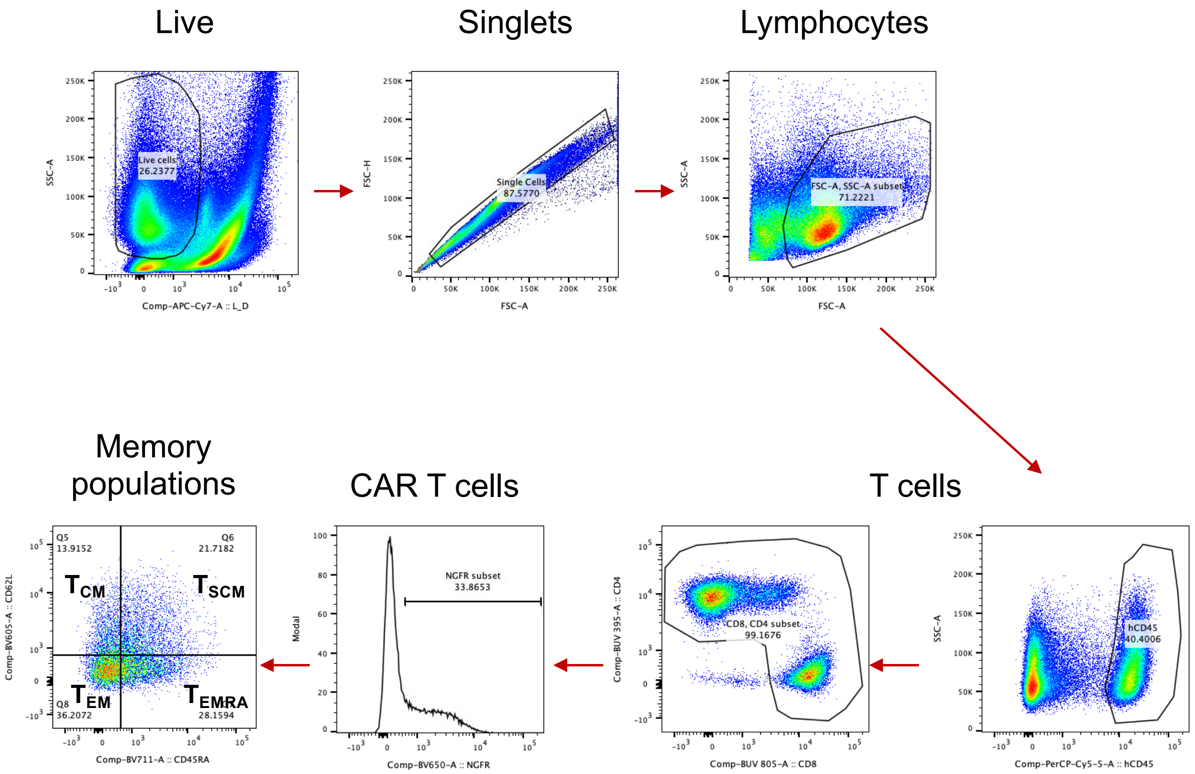

Supplementary Figure 1: Gating strategy for the analysis of CAR T cells from murine spleen by flow cytometry (Figure 3 **g-i**). Dead cells were excluded by staining with eBioscience™ Fixable Viability Dye eFluor™ 780. Cell doublets were excluded by gating cells on the diagonal of FSC-A/FSC-H. T cells were defined as hCD45^{+}, subsequently gated by hCD4 and hCD8. Memory subpopulations were defined based on the frequency of CD62L+/- CD45RA+/- within the NGFR^{+} population.

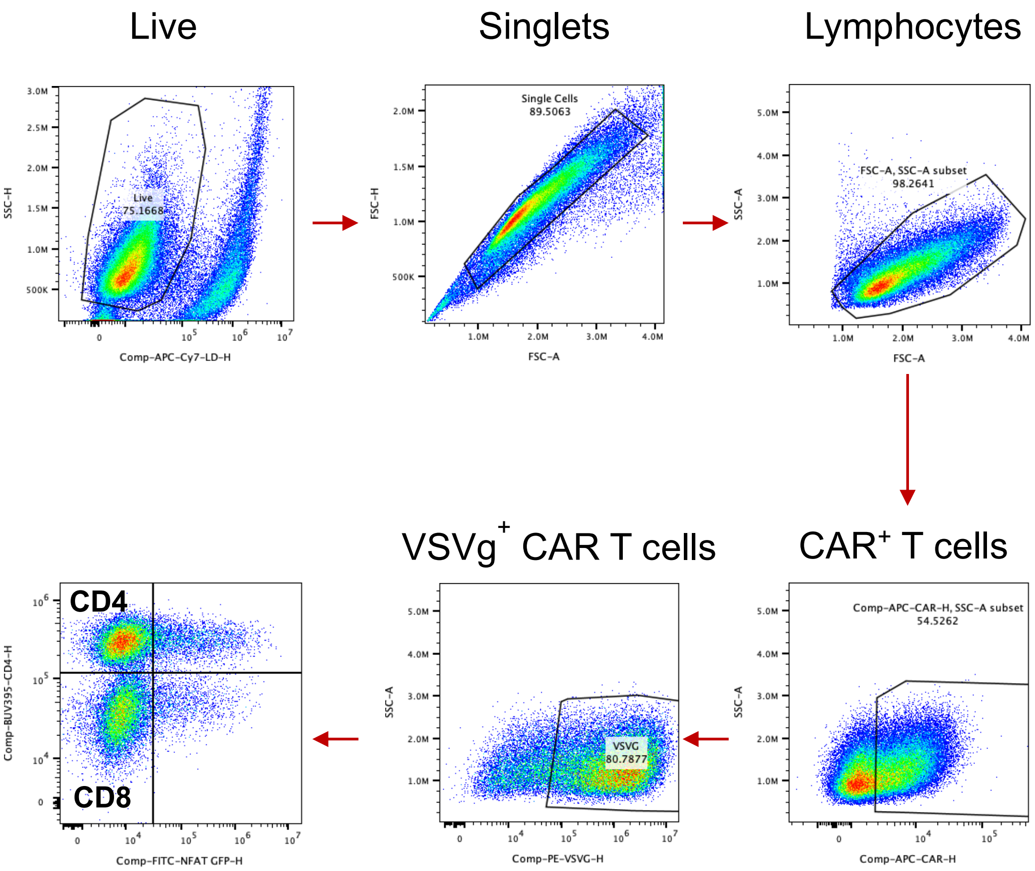

Supplementary Figure 2: Gating strategy for the analysis of the NFAT-GFP T cell activation assay (Figure 6 **c-d**, Extended Data Figure 5 **b**). Dead cells were excluded by staining with eBioscience™ Fixable Viability Dye eFluor™ 780. Cell doublets were excluded by gating cells on the diagonal of FSC-A/FSC-H. T cells expressing CAR and VSV-G were identified, and NFAT-induced GFP expression was assessed within $CD4^+$ and $CD8^+$ subsets.
