# Supplementary material for: Engineering T cells with a membrane-tethered version of SLP-76 overcomes antigen-low resistance to CAR T cell therapy
Source: Nat Cancer. 2025 Oct 23;6(12):1940–54. doi: 10.1038/s43018-025-01056-4 (PMC12727510; doi:10.1038/s43018-025-01056-4)
Supplement: Supplementary file 2 — Reporting Summary [file 43018_2025_1056_MOESM2_ESM.pdf]

nature portfolio

Corresponding author(s): Robbie G. Majzner

Last updated by author(s): Jul 26, 2025

Reporting Summary

Nature Portfolio wishes to improve the reproducibility of the work that we publish. This form provides structure for consistency and transparency in reporting. For further information on Nature Portfolio policies, see our Editorial Policies and the Editorial Policy Checklist.

Statistics

For all statistical analyses, confirm that the following items are present in the figure legend, table legend, main text, or Methods section.

n/a	Confirmed	
☐	☒	The exact sample size (*n*) for each experimental group/condition, given as a discrete number and unit of measurement
☐	☒	A statement on whether measurements were taken from distinct samples or whether the same sample was measured repeatedly
☐	☒	The statistical test(s) used AND whether they are one- or two-sided<br>*Only common tests should be described solely by name; describe more complex techniques in the Methods section.*
☒	☐	A description of all covariates tested
☐	☒	A description of any assumptions or corrections, such as tests of normality and adjustment for multiple comparisons
☐	☒	A full description of the statistical parameters including central tendency (e.g. means) or other basic estimates (e.g. regression coefficient) AND variation (e.g. standard deviation) or associated estimates of uncertainty (e.g. confidence intervals)
☐	☒	For null hypothesis testing, the test statistic (e.g. *F*, *t*, *r*) with confidence intervals, effect sizes, degrees of freedom and *P* value noted<br>*Give P values as exact values whenever suitable.*
☒	☐	For Bayesian analysis, information on the choice of priors and Markov chain Monte Carlo settings
☒	☐	For hierarchical and complex designs, identification of the appropriate level for tests and full reporting of outcomes
☒	☐	Estimates of effect sizes (e.g. Cohen's *d*, Pearson's *r*), indicating how they were calculated

Our web collection on statistics for biologists contains articles on many of the points above.

Software and code

Policy information about availability of computer code

Data collection	FACSDiva ver 8.0.1 (BD Biosciences): Flow cytometric data acquisition<br>NovoExpress ver 1.6.2 (Agilent): Flow cytometric data acquisition<br>Gen5 ver 2.00.18 (BioTek): Colorimetric ELISA quantification for cytokine release<br>IncuCyte S3 ver 2019B Rev2 Software (Sartorius): Cytotoxicity assay<br>LivingImage ver 4.7.3 (Perkin Elmer): In vivo bioluminescence imaging<br>Aura ver 4.0.7 (Spectral Instrument Imaging): In vivo bioluminescence imaging
Data analysis	FlowJo ver 10.10: Flow cytometric data analysis<br>Microsoft Excel ver 16.85: Bulk data analysis<br>GraphPad Prism ver 10.2.3: Graphs generation and statistical analysis<br>SnapGene ver 7.2.1: DNA sequences analysis and molecular cloning<br>Proteome Discoverer ver 2.3: Data processing and target decoy search of mass spectrometry data<br>CellRanger v.6.0: demultiplexing and alignment of sequencing reads to host reference genome<br>mgatk v.0.6.2: demultiplexing of multiple donors using mitochondrial DNA genotypes<br>Seurat v.5.1: cell type identification, differential gene expression analyses and visualization<br>R v.4.2.2: statistical, mass spectrometry and scRNA seq data analysis

For manuscripts utilizing custom algorithms or software that are central to the research but not yet described in published literature, software must be made available to editors and reviewers. We strongly encourage code deposition in a community repository (e.g. GitHub). See the Nature Portfolio guidelines for submitting code & software for further information.

Data

Policy information about availability of data

All manuscripts must include a data availability statement. This statement should provide the following information, where applicable:

- Accession codes, unique identifiers, or web links for publicly available datasets
- A description of any restrictions on data availability
- For clinical datasets or third party data, please ensure that the statement adheres to our policy

All mass spectrometry data have been deposited to the ProteomeXchange Consortium via the PRIDE partner repository (dataset identifier: PXD053205).
The scRNA-seq datasets have been deposited in the NCBI Gene Expression Omnibus (GEO) and are accessible through the GEO series accession number GSE270399

Research involving human participants, their data, or biological material

Policy information about studies with human participants or human data. See also policy information about sex, gender (identity/presentation), and sexual orientation and race, ethnicity and racism.

Reporting on sex and gender	*Use the terms sex (biological attribute) and gender (shaped by social and cultural circumstances) carefully in order to avoid confusing both terms. Indicate if findings apply to only one sex or gender; describe whether sex and gender were considered in study design; whether sex and/or gender was determined based on self-reporting or assigned and methods used. Provide in the source data disaggregated sex and gender data, where this information has been collected, and if consent has been obtained for sharing of individual-level data; provide overall numbers in this Reporting Summary. Please state if this information has not been collected. Report sex- and gender-based analyses where performed, justify reasons for lack of sex- and gender-based analysis.*
Reporting on race, ethnicity, or other socially relevant groupings	*Please specify the socially constructed or socially relevant categorization variable(s) used in your manuscript and explain why they were used. Please note that such variables should not be used as proxies for other socially constructed/relevant variables (for example, race or ethnicity should not be used as a proxy for socioeconomic status). Provide clear definitions of the relevant terms used, how they were provided (by the participants/respondents, the researchers, or third parties), and the method(s) used to classify people into the different categories (e.g. self-report, census or administrative data, social media data, etc.) Please provide details about how you controlled for confounding variables in your analyses.*
Population characteristics	Buffy coats, leukopaks or leukocyte reduction system (LRS) chambers were obtained from consenting healthy donors through the Stanford Blood Center under an institutional review board (IRB)-exempt protocol.
Recruitment	*Describe how participants were recruited. Outline any potential self-selection bias or other biases that may be present and how these are likely to impact results.*
Ethics oversight	*Identify the organization(s) that approved the study protocol.*

Note that full information on the approval of the study protocol must also be provided in the manuscript.

Field-specific reporting

Please select the one below that is the best fit for your research. If you are not sure, read the appropriate sections before making your selection.

☒ Life sciences ☐ Behavioural & social sciences ☐ Ecological, evolutionary & environmental sciences

For a reference copy of the document with all sections, see nature.com/documents/nr-reporting-summary-flat.pdf

Life sciences study design

All studies must disclose on these points even when the disclosure is negative.

Sample size	Sample size calculations were not performed. Sample sizes were determined based on prior laboratory experience with well-established, previously published models (1,2), and are reported for each experiment.<br>1. Majzner, R.G., et al., Tuning the Antigen Density Requirement for CAR T-cell Activity. Cancer Discov, 2020. 10(5): p. 702-723.<br>2. Tousley, A.M., et al., Co-opting signalling molecules enables logic-gated control of CAR T cells. Nature, 2023. 615(7952): p. 507-516.
Data exclusions	No data was excluded from analysis.
Replication	In vitro and in vivo experiments were reproduced in technical and biological replicates as stated in figure legend.
Randomization	For in vivo studies, mice were randomized to ensure equal mean tumor burden before T cell treatment.
Blinding	For in vivo studies, the technician performing tumor and T cell injections was blinded to the treatment and expected outcomes. No blinding methods were used for the other experiments due to personnel availability to accommodate such situations.

Reporting for specific materials, systems and methods

We require information from authors about some types of materials, experimental systems and methods used in many studies. Here, indicate whether each material, system or method listed is relevant to your study. If you are not sure if a list item applies to your research, read the appropriate section before selecting a response.

Materials & experimental systems

n/a	Involved in the study
☐	☒ Antibodies
☐	☒ Eukaryotic cell lines
☒	☐ Palaeontology and archaeology
☐	☒ Animals and other organisms
☒	☐ Clinical data
☒	☐ Dual use research of concern
☒	☐ Plants

Methods

n/a	Involved in the study
☒	☐ ChIP-seq
☐	☒ Flow cytometry
☒	☐ MRI-based neuroimaging

Antibodies

Antibodies used

From Abcam:

VSV-G (FITC, polyclonal, ab3863, 1:100)
VSV-G (biotin, polyclonal, ab34774, 1:100)

From BD Biosciences:

NGFR (BV421, clone C40-1457, catalog: 562562, 1:200)
CD4 (BUV 395 clone SK3, catalog: 563552, 1:100)
CD8 (BUV 805, clone SK1, catalog: 612889, 1:200)
CD62L (BV605, clone DREG-56, catalog: 562719, 1:100)
CD45RA (BV711, clone HI100, catalog: 563733, 1:100)
PLCg1 (Alexa Fluor 647, clone 10, catalog: 558565, 1:12.5)
SLP-76 (Alexa Fluor 647, clone H3, catalog: 560057, 1:12.5)
BD QuantiBRITE™ PE beads (catalog: 340495)

From Biolegend:
HA (Pacific Blue, clone 16B12, catalog: 901526, 1:100)
Streptavidin (PE, catalog: 405245, 1:100)
CD45 (PerCP/Cy5.5, clone 2D1, catalog: 304028, 1:100)
CD19 (APC or PE, clone HIB19, catalog: 302212 and 302208, 1:50)
CD22 (APC, clone HIB22, catalog: 302510; PE, clone S-HCL-1, catalog: 363503, 1:50)
HER2 (PE–Cy7 or PE clone 24D2, catalog: 324414 and 324405, 1:50)
ROR1 (PE–Cy7, clone 2A2, catalog: 357808, 1:50)
BCMA (PE, clone 19F2, catalog: 357504, 1:100)
Lck (Alexa Fluor 647, clone Lck-01, catalog: 628304, 1:200)
ZAP-70 (Alexa Fluor 647, clone A16043B, catalog: 693508, 1:100)

eBioscience

Fixable Viability Dye (eFluor 780, catalog: 65-0865-14, 1x)

Custom

Anti-CD19 CAR (clone FMC63): Sourced from Genscript via custom preparation, conjugated using the DyLight 650 Labeling kit (catalog: 84536)

R&D
LAT (Alexa Fluor 647, clone 661002, catalog: FAB63341R, 1:200)
Recombinant Human Siglec-2/CD22 Fc Chimera Protein (catalog: 1968-SL-050)
Recombinant Human ErbB2/Her2 Fc Chimera Protein (catalog: 1129-ER-050)
Recombinant Human BCMA/TNFRSF17 Fc Chimera Protein (catalog: 193-BC-050)
Recombinant Human ROR1 Fc Chimera Protein (catalog: 9490-RO-050)
Recombinant proteins were conjugated using the DyLight 650 Labeling kit (catalog: 84536)

Validation

All antibodies were validated by the manufacturer, as documented on the manufacturer's website using the provided catalog numbers. The anti-CD19 CAR idiotype antibody and the staining with the recombinant proteins were validated in house using untransduced T cells as negative biologic control (used as 1:400 dilution).

Eukaryotic cell lines

Policy information about cell lines and Sex and Gender in Research

Cell line source(s)	Nalm6-GL was originally provided by Steve Grupp (University of Pennsylvania, Philadelphia, PA), originally obtained from American Type Culture Collection (ATCC, Manassas). The OPM-2-GFP Luciferase multiple myeloma cell line was obtained from Eric Smith (Dana-Farber Cancer Institute). 293GP line was provided by the Surgery Branch (NCI).
Authentication	All cell lines were previously authenticated by STR fingerprinting prior to their use in the manuscript. Antigen expression was routinely verified via flow cytometry.
Mycoplasma contamination	All cell lines tested negative for mycoplasma using the MycoAlert Mycoplasma Detection Kit (Lonza).
Commonly misidentified lines (See ICLAC register)	No commonly misidentified lines were used.

Animals and other research organisms

Policy information about studies involving animals; ARRIVE guidelines recommended for reporting animal research, and Sex and Gender in Research

Laboratory animals	4- to 10-week-old male or female NOD/SCID/IL2Rg (NSG, NOD.Cg-Prkdcscid Il2rgtm1Wjl/SzJ) mice were used for all in vivo experiments. Mice were housed at 22°C and 50% humidity with a 12-hour light/12-hour dark cycle.
Wild animals	The study didn't involve wild animals.
Reporting on sex	Relatively equal numbers of male and female mice (sex matched for each experiment) were used for in vivo experiments.
Field-collected samples	The study did not involve field-collected samples.
Ethics oversight	Animal studies were carried out according to Stanford Institutional Animal Care and Use Committee-approved protocol (protocol 33698).

Note that full information on the approval of the study protocol must also be provided in the manuscript.

Plants

Seed stocks	*Report on the source of all seed stocks or other plant material used. If applicable, state the seed stock centre and catalogue number. If plant specimens were collected from the field, describe the collection location, date and sampling procedures.*
Novel plant genotypes	*Describe the methods by which all novel plant genotypes were produced. This includes those generated by transgenic approaches, gene editing, chemical/radiation-based mutagenesis and hybridization. For transgenic lines, describe the transformation method, the number of independent lines analyzed and the generation upon which experiments were performed. For gene-edited lines, describe the editor used, the endogenous sequence targeted for editing, the targeting guide RNA sequence (if applicable) and how the editor was applied.*
Authentication	*Describe any authentication procedures for each seed stock used or novel genotype generated. Describe any experiments used to assess the effect of a mutation and, where applicable, how potential secondary effects (e.g. second site T-DNA insertions, mosiacism, off-target gene editing) were examined.*

Flow Cytometry

Plots

Confirm that:

☒ The axis labels state the marker and fluorochrome used (e.g. CD4-FITC).

☒ The axis scales are clearly visible. Include numbers along axes only for bottom left plot of group (a 'group' is an analysis of identical markers).

☒ All plots are contour plots with outliers or pseudocolor plots.

☒ A numerical value for number of cells or percentage (with statistics) is provided.

Methodology

Sample preparation	Sample preparation is outlined in the methods. Briefly, cells were washed with 2% FBS in PBS.<br>Cells were subsequently stained with cell surface antibodies for 10 minutes at room temperature protected from light. Cells were then washed with 2% FBS in PBS and analyzed. For intracellular staining, the manufacturer's protocol of the Foxp3/ Transcription Factor Staining Buffer Set (eBioscience) was followed. Cells were sorted using a 100 micron nozzle.

Instrument	BD Fortessa and Agilent NovoCyte Quanteon and Penteon flow cytometers.
Software	FACSDiva ver 8.0.1 (BD Biosciences) and NovoExpress ver 1.6.2 (Agilent) for data collection. FlowJo ver 10.10 (BD Biosciences) for data analysis.
Cell population abundance	For in vitro analysis of tumor or T cells, abundance was >95%. For ex vivo analysis, samples had varied abundance depending on experiments.
Gating strategy	If a Live/Dead staining was used, cells were first gated for live cells (less stained population). Cells were then gated for single cells (FSC-A/FSC-H), lymphocyte population (FSC-A/SSC-A) and relevant surface markers.<br>Exemplary gating strategies are provided in Supplementary Figures, FMOs were used to determine the frequency of CD62L+/- CD45RA+/- memory T cell populations.

☒ Tick this box to confirm that a figure exemplifying the gating strategy is provided in the Supplementary Information.
